# Systematic investigation of interindividual variation of DNA methylation in human whole blood

**DOI:** 10.1186/s13059-026-04021-1

**Published:** 2026-03-05

**Authors:** Olivia A. Grant, Meena Kumari, Leonard Schalkwyk, Nicolae Radu Zabet

**Affiliations:** 1 School of Life Sciences, University of Essex, Wivenhoe Park, Colchester, CO4 3SQ UK; 2 Institute of Social and Economic Research, University of Essex, Wivenhoe Park, Colchester, CO4 3SQ UK; 3 Blizard Institute, Barts and The London School of Medicine and Dentistry, Queen Mary University of London, 4 Newark St, London, E1 2AT UK; 4 Institute of Psychiatry, Psychology & Neuroscience, Kings College London, Denmark Hill, London, SE5 9RS UK; 5 Centre for Epigenetics, Queen Mary University of London, 4 Newark St, London, E1 2AT UK

**Keywords:** DNA methylation, Epigenetic interindividual variation, Cis-regulatory regions, Transcription factors

## Abstract

**Background:**

Interindividual genetic variability is well characterised, but we still lack a complete catalogue of loci displaying variable and stable epigenetic patterns.

**Results:**

Here, we report a catalogue of stable and variable interindividual DNA methylation sites in human whole blood by analysing the DNA methylation patterns in 3642 individuals from a representative cohort for the British population using the IlluminaEPIC array. Our results show that 34,972 CpGs display variable methylation levels (VMPs) and 41,216 CpGs display stable methylation. Human whole blood is a widely used tissue in epigenetic research, particularly in Epigenome-Wide Association Studies, due to its accessibility and its ability to provide insights into systemic biological processes and disease mechanisms. This catalogue is a useful resource for interpretation of results when associating epigenetic signals to phenotypes. VMPs are highly enriched in CpG shores, enhancers and intergenic regions and approximately half of the VMPs are under genetic control. Our results also showed that trans mQTL-mCpG pairs (that is a SNP and CpG located > 500bp apart) are often located in the same TAD or connected by chromatin loops. A subset of these VMPs (784) are classified as putative epialleles and there is a link between some of these epialleles located in regulatory regions and gene expression.

**Conclusions:**

Our study provides of a comprehensive and reliable catalogue of CpG sites displaying variable interindividual DNA methylation across the human epigenome.

**Supplementary Information:**

The online version contains supplementary material available at 10.1186/s13059-026-04021-1.

## Background

The combination of genetic, epigenetic and environmental variation between individuals is responsible for the large diversity observed in human phenotypes. Large efforts have previously been made to characterise genetic variation in humans, with important advances such as the characterisation of millions of single nucleotide polymoprhisms (SNPs) [1, 2], yet detailed catalogues of epigenetic variation are still not complete.

The need for these efforts are becoming increasingly clear, as research suggests that changes to DNA sequence and exposure to known environmental factors are unable to account for some phenotypic differences that can be observed amongst a population. This has been highlighted by studies in genetically identical organisms, commonly involving mono-zygotic twins but also some inbred animal studies [3]. Monozygotic twins in almost all cases, are identical in appearance, however are often discordant for particular diseases or phenotypes. Usually, this discordance has been attributed to differences in environmental exposures which can have widespread and significant effects on human health. It is becoming increasingly accepted that epigenetic mechanisms may explain these findings in twin and animal studies. Thus, recent studies have identified epigenetic differences between monozygotic twins who are discordant for particular diseases such as amyotrophic lateral sclerosis, psoriasis and neurofibromatosis, thus suggesting that epigenetic variation could in fact explain differences in phenotype [4–6].

In understanding the intricate mechanisms underlying epigenetic variation, three main epigenetic marks play pivotal roles: histone modifications [7], RNAs (such as microRNAs and long non-coding RNAs) [8], and DNA methylation [9]. DNA methylation, on the other hand, is a key epigenetic modification involving the addition of methyl groups to cytosine residues in the DNA molecule, often occurring at CpG dinucleotides [10]. Recognizing the significance of understanding the interplay between genetics and epigenetics in shaping phenotypic diversity, our study focuses specifically on DNA methylation. This choice is motivated by the growing body of evidence suggesting that alterations in DNA methylation patterns contribute significantly to phenotypic differences, particularly in instances where genetic and environmental factors alone cannot account for observed disparities [11].

To this effect, previous studies have aimed to characterise a catalogue of loci showing highly variable DNA methylation in a range of tissues including peripheral blood, cord blood, saliva, placenta and colon [12–17]. Nevertheless, this is a difficult task due to the dynamic nature of the epigenome. Whilst genetic variation is minimal within individuals (intraindividual) and extensive between individuals (interindidvidual), DNA methylation variation is extensive both within and between individuals. This is because individuals within a population can vary for a wide variety of reasons (known examples include stress levels, age, sex and smoking status) all of which in turn, can cause variation in the epigenome. Additionally, these varying patterns may differ between different tissues and cell types, unlike genetic variation.

The Human Epigenome project is an important resource for mapping the human epigenome and some of their efforts were in fact directed towards characterising interindividual variation [18]. However, these efforts were focused mainly on an approximately 4 Mb region of the genome called the Major Histocompatibility Complex (MHC) rather than genome wide. One important result they found is that DNA methylation variability is often tissue dependent, as the loci they investigated did not show concordant variation across tissues. Furthermore, these highly variable amplicons were mostly intragenic [18], a finding which was also observed in another independent study identifying interindividual Differentially Methylated Regions (DMRs) in monocytes [19]. Additional work in human germ cells also supports this idea and further detected a large degree of variation within promoter CpG islands and pericentromeric satellites [20].

A recent study identified variably methylated regions (VMRs), defined as clusters of CpGs with high interindividual epigenetic variation [14]. It also found that these regions were enriched at enhancers and 3’UTRs and imprinted loci, which suggests that they may play a functional role in gene expression or biological function.

Additionally, it is now also well known that DNA methylation is strongly influenced by genetic variation. A clear example is provided by the evidence that a rare polymorphism at DNMT3L, leads to significant DNA hypomethylation in subtelomeric regions of the genome [21]. Nevertheless, DNA methylation can also impact genetic variation and one of the simplest cases is deamination of a methylated cytosine, by which a methylated cytosine gets converted to a thymine. In this case, the lack of cytosine would result in an absence of DNA methylation, so the variation would result in either no DNA methylation or a fully methylated position [22].

Most evidence showing a relationship between genetic variation and epigenetic variation arises from work characterising the methylation quantitative trait loci (mQTLs), which are genetic variants that influence CpG methylation [23]. mQTLs have previously been characterised in brain, blood, lung and adipose tissue [24–29]. Additionally, these methylation quantitative trait loci may also overlap variants that associate with gene expression levels (eQTLs). Thus, the research linking these epigenetic signatures to genotypes is vital to provide more mechanistic insights into the interplay between genetics, disease and epigenetic variation.

In this study, we identified a robust catalogue of loci within the human genome with either high interindividual variability or high interindividual stability in DNA methylation in whole blood. We do this by leveraging two large datasets from Understanding Society [30] investigating 850,000 CpG sites among a total of over 3.6*K* individuals (discovery dataset, $n =1,171$, and validation dataset, $n=2,471$). To our knowledge, this is the largest study using the Illumina EPIC BeadChip (allowing for interrogation of 850,000 sites across the genome) to investigate variability and stability in DNA methylation at CpG sites in whole blood. Other studies used the 450 K array [14, 17], which has approximately half of the CpGs investigated here, and provides a lower coverage of distal regulatory elements beyond promoters and CpG islands, or performed bisulfite sequencing which suffers from lower sample size due to higher costs [31, 32]. It is important to highlight that the data utilised in this study is derived from a representative cohort for the UK population which may limit the generalisability of the findings to other populations. Nonetheless, this is one of the largest cohort that is not disease or phenotype specific.

## Results

### Stable and variable methylated probes show distinct regulatory enrichment

**Fig. 1:**
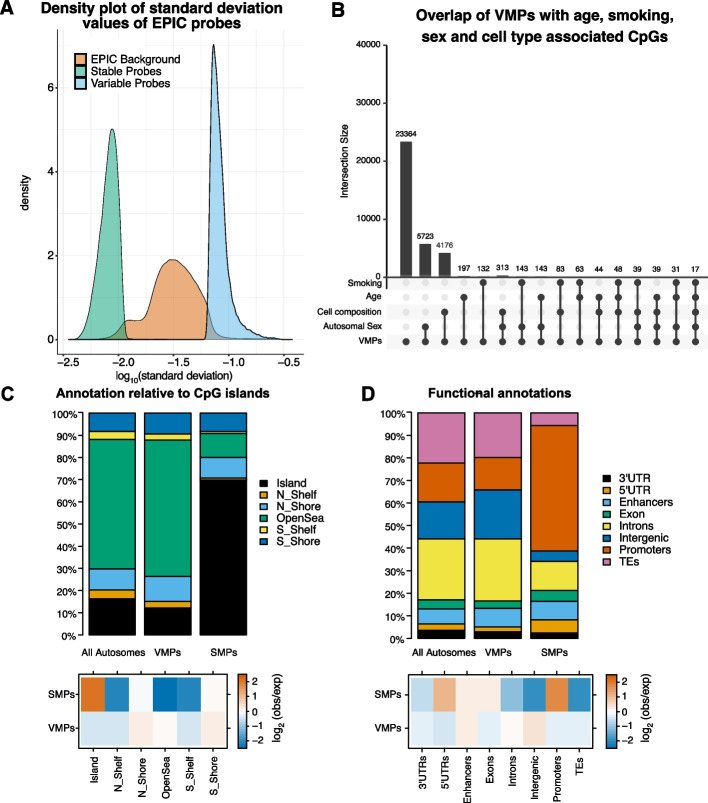
Identification and annotation of variably methylated probes (VMPs) and stably methylated probes (SMPs). **A** Density plot representing the standard deviation values of methylation across our samples for VMPs, SMPs and the autosomal EPIC background. **B** Upset plot displaying the number of VMPs that overlap with known autosomal sex, smoking, cell type and age associated CpGs. We also considered probes that are not identified as VMPs when adjusting for cell type composition. **C**, **D** The annotation of VMPs ($n= 34,972$) and SMPs ($n=40,288$) relative to: (**C**) CpG islands (**D**) genomic features. Bottom panel shows the $log_2 (observed/expected)$ annotations based on the autosomal EPIC background of the different annotations. Note that all autosomes include the EPIC background, VMPs and SMPs, with the majority (90%) consisting of the EPIC background

We characterised interindividual variation of DNA methylation in whole blood using a discovery and validation approach using two human sample sets ($n=1,171$ and $n=2,471$, respectively). This analysis was performed on the Illumina EPIC array, which covers 850,000 sites across the human genome. Sites which were known SNP probes, cross hybridizing probes or sex chromosome linked probes were removed from our analysis. Thus, after quality checks and data processing, a total of 747,302 CpGs were included in our analysis (see Material and Methods). We identified 34, 972 CpGs that display highly variable methylation levels in both our discovery and validation data sets across our population, which we termed variably methylated probes (VMPs) and 41, 216 CpGs that show robust stable methylation state (i.e., low interindividual variability) in both our discovery and validation data sets (with majority being either unmethylated or methylated in all individuals), which we termed stably methylated probes (SMPs); see Fig. 1A and Additional file 1: Fig. S1A, B; Additional files 2 and 3). Approximately 1% of SMPs (553 probes, 3.9 times more than expected by chance, Fisher’s exact test *p*-value = $5\times 10^{-4}$) are in non-CpG context which are expected to be unmethylated and show no interindividual variation in methylation, as we would expect in non stem cells [33]. Half of the VMPs and 20% of the SMPs are EPIC specific, which means they could not have been previously identified on studies that considered only 450 K array datasets (Additional file 1: Fig. S1C, D). Interestingly, VMPs showed predominantly intermediate methylation levels, in contrast to SMPs that displayed either low or high methylation (see Additional file 1: Fig. S2A-C).

Several previous studies use analysis of duplicate arrays to classify probes by reliability on the basis of an interclass correlation (ICC) threshold [34–36]. Out of 34, 972 VMPs, only 1, 515 (representing only 4.3% of VMPs) had an ICC lower than 0.4 and could be labelled as unreliable (Additional file 4). Nevertheless, 82% of SMPs (33, 776 out of 41, 216 SMPs) had an ICC lower than 0.4 and could be labelled as unreliable (Additional file 5). For these SMPs, we found very low interindividual variation in our dataset (over 3.6*K* individuals), which accounts for both biological and technical variation. This highlights that ICC might not be the most appropriate method to describe *reliability* of probes: probes whose DNA methylation state is reliably measured and does not vary in a given cohort are labelled *unreliable* according to ICC. Thus, in the downstream analysis we did not filter out probes with lower reported ICC.

Given that interindividual variation in DNA methylation may relate to environmental differences, we investigated the overlap of VMPs and SMPs with CpGs associated with phenotypes that are known to have strong associated epigenetic signatures, namely: age, smoking and sex. We found that despite VMPs having a higher overlap with known sex, smoking or age associated CpGs than SMPs,there were still a large proportion of VMPs that did not overlap with CpGs associated with these phenotypes indicating that these could be under the control of other environmental or genetic factors or variable methylation could in fact have stochastic nature (see Fig. 1B and Additional file 1: Fig. S2D).

Blood cell type composition varies among individuals, and each cell type has a distinctive DNA methylation profile, so cell type composition is one potential cause of DNA methylation variation. Only 42 of the 450 CpGs previously identified as optimal for distinguishing blood cell types [37] overlap with our VMPs and none with our SMPs, which supports that the observed variability is not primarily driven by cell composition. Those 450 probes do not consist of all of the cell type differences, but they were chosen as optimal for distinguish cell types and so one would expect that majority of those probes are in the VMP list if cell type is an important driver of VMPs. Furthermore, we have also computed VMPs and SMPs with a model adjusting for cell type proportions; see Materials and Methods. Our analysis identified that 4,759 VMPs (13.6%) and 4,239 SMPs (10%) would be lost if we include cell type proportions as covariates, indicating that less than 15% of the inter individual variable probes in DNA methylation can be attributed to cell type proportions (see Fig. 1B, Additional file 1: Fig. S2D and Additional files 6 and 7). In other words, cellular heterogeneity was directly quantified as part of our study, and we showed that it contributes to only a small percentage ($< 15\%$) of the epigenetic variation observed.

Next, we investigated if the VMPs and SMPs are assigned to either housekeeping or tissue specific genes. Unsurprisingly, our results show that SMPs are usually enriched at housekeeping genes, while VMPs at tissue specific genes (see Additional file 1: Fig. S2E).

We also investigated the distance between neighbouring VMPs or SMPs and found that on average VMPs are $64\ Kb$ apart, while SMPs are $30\ Kb$ apart (see Additional file 1: Fig. S2F), with 70% of the VMPs being further $2,544\ bp$ and 70% of the SMPs being closer than $1,963\ bp$. To help us gain more insight into the functional role of these variably and stably methylated probes, we characterised their location with respect to CpG islands and functional regions. We found that VMPs are enriched at CpG shores and depleted at CpG islands and CpG shelves compared with distribution of all EPIC probes within those regions, $log_2(observed/expected)$ (see Fig. 1C). In contrast, we observed that SMPs were highly enriched in CpG islands and depleted at CpG shelves and open sea regions. Furthermore, VMPs were enriched at intergenic regions, introns and enhancers, but depleted at UTRs, promoters and transposable elements (see Fig. 1D). On the other hand, SMPs were enriched at 5’UTRs and promoter regions of the genome, and showed slight enrichment at enhancers. Altogether, we found that SMPs are located at housekeeping promoters and VMPs are located in regulatory regions (enhancers and intergenic regions) of tissue specific genes.

Imprinted control regions display high methylation on either maternal or paternal copies of the DNA with the other copy being unmethylated [38]. Given the specific signatures of methylation at these imprinted regions, we also investigated whether variable probes were enriched at imprinted regions of the genome. While only one single stably methylated probe overlaps imprinted regions, we found 242 VMPs that overlap an imprinted gene, which is higher than we would expect by chance compared to the EPIC array background (Permutation test, *p*-value 0.001); see Additional file 1: Fig. S2G.

### VMPs and SMPs located at promoters and enhancers are enriched for transcription factor motifs

**Fig. 2:**
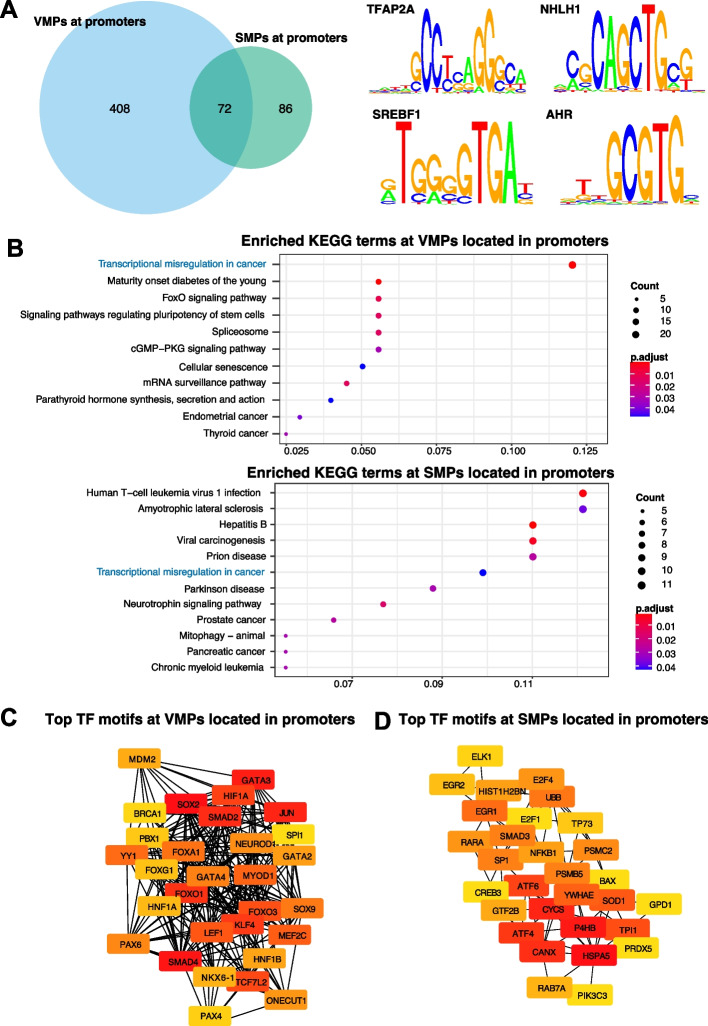
Transcription factor motif enrichment for VMPs and SMPs at promoters. **A** Overlap of enriched TF motifs for VMPs (blue) and SMPs (green). The top two motifs enriched at VMPs were TFAP2A and NHLH1 and at SMPs were SREBF1 and AHR. **B** KEGG analyses for the significantly enriched TF motifs at VMPs and SMPs. Common terms are highlighted with blue colour. **C**, **D** Sub networks of the top 30 enriched TF motifs at (**C**) VMPs and (**D**) SMPs. Node colour represents the degree of connectivity. The scale from red to yellow represents the top 30 enriched TF motif rank from 1–30, with red indicating highest degree and yellow indicating lowest degree

DNA methylation has been shown to impact binding of TFs to DNA [39, 40], which downsteam can impact gene regulation. To investigate this, we performed transcription factor (TF) binding motif enrichment and gene ontology analyses for our VMPs and SMPs located at promoters and enhancers specifically. First, we evaluated whether the VMPs and SMPs located at promoters were enriched in motifs for TFs (50 bp window around the CpG). For the VMPs we found 408 unique enriched TF motifs (these 408 motifs were specific to VMPs only), with strongest evidence for TFAP2A and NHLH1 (Fig. 2A). For SMPs, we identified 86 unique enriched TFs (specific only to SMPs), including SREBF1 and AHR (Fig. 2A). Similarly, for VMPs and SMPs located at enhancers, we identified 376 uniquely enriched TF motifs at VMPs and 74 enriched at SMPs (Additional file 1: Fig. S3A). The most strongly enriched TFs at enhancers differed to those at promoters, with ATF2 and FOS at VMPs and NNT and ODC1 at SMPs (see Additional file 1: Fig. S3A). The fact we found different TF motifs enriched at VMPs compared to SMPs, including some that show DNA methylation binding sensibility, indicates that variation in DNA methylation at these CpGs would impact binding of those TFs and consequently could potentially have an impact on gene regulation.

To investigate whether the transcription factor motifs were enriched for terms related to biological processes or pathways, we performed pathway analyses using the GO and KEGG databases. We identified several enriched KEGG pathways for the TF motifs enriched at VMPs in promoters, spanning a wide range of processes such as transcriptional misregulation in cancer, FoxO signalling pathways and signalling pathways regulating pluripotency of stem cells (see Fig. 2B). We also identified several enriched terms for the TF motifs enriched at SMPs in promoters such as viral carcinogenesis, prion disease and Parkinson disease (see Fig. 2B). Interestingly, for those TF motifs enriched at VMPs in enhancers, we find similar pathways, such as transcriptional misregulation in cancer (see Additional file 1: Fig. S3B).

We then used cytohubba (a cytoscape tool) to identify if any of these transcription factors are regulatory hubs controlling many genes. For the TF motifs enriched at VMPs within promoters we identified MAPK1, GATA3 and SOX2 and other genes to be hub TFs in the network (see Fig. 2C). Moreover, for those TFs enriched at SMPs within promoters we identified CYCS, P4HB and HSPA5 to be hubs in this network (see Fig. 2D). Similar to our TF motif enrichment analyses, we also found a large overlap between the hub TFs identified at promoters and enhancers for both VMPs and SMPs (see Additional file 1: Fig. S3C, D). Interestingly, both VMPs within enhancers and promoters display enrichment of motifs for JUN, HIF1A and FOS. TET enzymes which are involved in active demethylation, display sequence preferences for these motifs, which indicates a potential mechanistic link between the enrichment of these TFs at VMPs and changes in DNA methylation [41].

### Approximately half of the VMPs are under genetic control

**Fig. 3:**
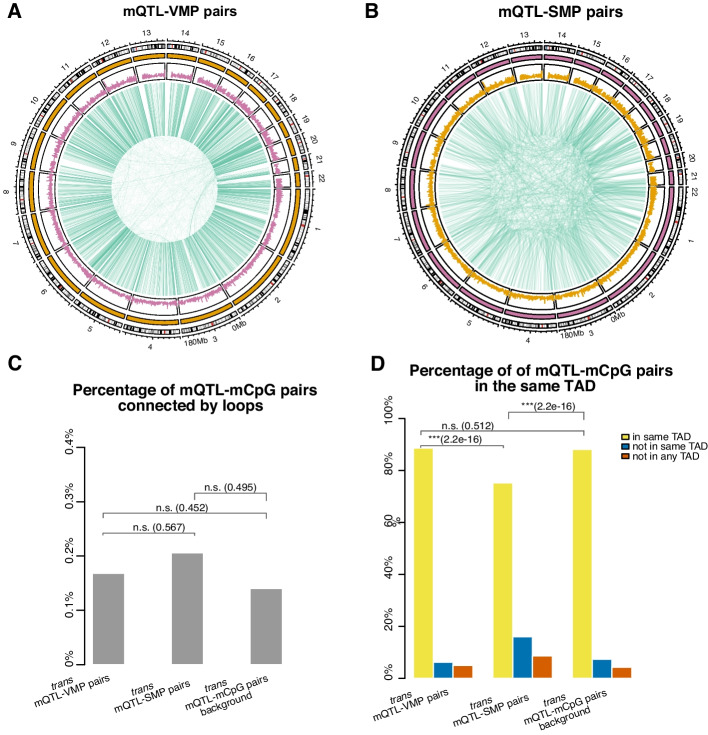
VMPs and SMPs associated with mQTLs. **A**, **B** Circo plot illustrating *cis* and *trans* (**A**) mQTL-VMP pairs and (**B**) mQTL-SMP pairs in whole blood. The outermost ring displays chromosome numbers and bands. The second ring represents CpG island density. The third ring represents associations between (**A**) mQTL-VMP pairs and (**B**) mQTL-SMP pairs (green lines). **C** Barplot representing the percentage of *trans* mQTL-VMP and mQTL-SMP pairs connected by chromatin loops. **D** Barplot representing the percentage of *trans* mQTL-VMP and mQTL-SMP pairs in the same topological associated domain. Fisher’s exact test was performed to determine statistical significance against the Illumina EPIC array background (*p*-value: n.s. $\ge$0.05, * <0.05, ** <0.01 and *** <0.001)

To gain insight into the mechanism that can explain the variability or stability at these CpGs, we also analysed a whole blood mQTL dataset [42]. Here, mQTLs are single-nucleotide polymorphisms (SNPs) that can affect DNA methylation levels at CpG sites [43]. Of the VMPs, 44.9% were associated with mQTLs and we call them mQTL-VMP pairs (see Fig. 3A and Additional file 8). We further categorised these into *cis* and *trans* mQTL-VMP pairs, where a *cis* mQTL-VMP pairs was defined when the SNP and CpG were less than or equal to 500bp away from one another, a *trans* mQTL-VMP pairs is defined when the SNP and CpG were more than 500bp away from one another. The intention in using such a small window (500 bp) was to distinguish differences driven by sequence variation in the CpG, the probe sequence or immediately flanking sequences at nucleosome scale (approximately 3 nucleosomes). Of the VMPs associated with mQTLs, 21% were *cis*, 79% were *trans*. One example is an mQTL-VMP pair located on chromosome 1 at the probe cg04315214 which is associated with two independent SNPs at the gene PRKCZ (FDR < 0.01). For SMPs, only 3.27% of the SMPs were associated with mQTLs and are called mQTL-SMP pairs (see Fig. 3B and Additional file 9), with 8.1% being *cis*, and 91.9% *trans*. An example of an mQTL-SMP pair is on chromosome 1 between cg04093404 and 4 SNPs at the gene TAS1R1. These results show that a large proportion of VMPs are associated with mQTLs indicating a link between the epigenetic variation at VMPs and genetic variation.

We also tested whether our mQTL-VMP and mQTL-SMP pairs were enriched in regulatory regions such as promoters or enhancers, however we found no significant enrichment in any particular regions that we would expect by chance. Mechanistically, the *trans* mQTL-VMP and mQTL-SMP pairs could be explained by the 3D chromatin organisation of the DNA, with SNPs and CpG sites that are distal on 1D, making 3D contacts. Thus, we hypothesised that the *trans* mQTL-VMP and mQTL-SMP pairs may occur at chromatin loops or topologically associated domains (TADs). Figure 3C shows that these *trans* mQTL-VMP and mQTL-SMP pairs were identified and categorised according to their chromosomal locations and potential involvement in 3D genomic structures.

**Fig. 4:**
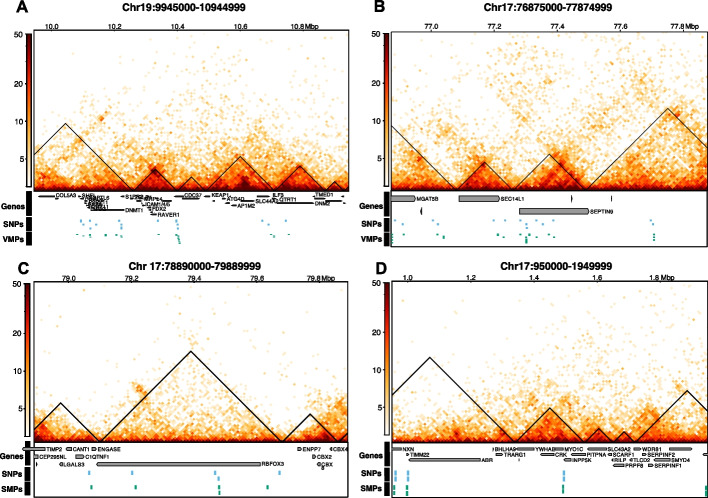
mQTL-VMP and mQTL-SMP pairs occupy the same topologically associated domains. **A** and **B** illustrate cases where the VMP and their associated SNP within a mQTL-VMP pair occupy the same TADs **C** and **D** illustrate cases where the SMP and their associated SNP within a mQTL-SMP pair occupy the same TADs. Hi-C interaction frequencies displayed as a two-dimensional heat map using *hicPlotTADs* tool from HiCExplorer [44]. x-axis indicates genomic position, y-axis represents the distance between the two genomic regions (in bp) and the colour the number of contacts between the two genomic regions (with darker colours indicating more contacts retrieved by HiC experiment and lighter colours representing fewer contacts). Black lines indicated TADs, green boxes indicate VMPs/SMPs, blue boxes indicate SNPs and grey boxes indicate genes which are appropriately labelled

Figure 3C shows that the *trans* mQTL-VMP, mQTL-SMP and mQTL-mCpG pairs were annotated to a very low percentage of loops. Chromatin loops call very strong interactions, but TADs identify regions that interact more with other regions inside the TAD than with regions outside the TAD thus allowing to capture finer connections between SNPs and VMPs. Our results show that a higher percentage of mQTL-VMP pairs occupy the same TAD than mQTL-SMP pairs and (Fisher’s exact test *p*-value < 0.05); see Fig. 3D. Nonetheless, we also found that approximately 90% of all *trans* mQTL-mCpG pairs, not only mQTL-VMP pairs, are located within the same TAD, showing that mQTL-VMP pairs are not located in the same TAD more than expected by chance (Fisher’s exact test *p*-value $= 0.5$). However, we note that our definition of *trans* is meant to investigate these pairs at nucleosome scale ($> 500\ bp$), but this is still very close (within 1 Mb). Examples of mQTL-VMP and mQTL-SMP pairs located at several different genes including DNMT1, SEPTIN9 and ILF3 are shown in Fig. 4.

Enrichment analyses of mQTL-VMP associated SNPs revealed several GO terms such as cell morphogenesis involved in neuron differentiation, small GTPase mediated signal transduction and cation transmembrane transporter activity (see Additional file 1: Fig. S4A). These SNPs were also enriched for few KEGG terms including Focal adhesion, axon guidance and cell ahesion molecules (see Additional file 1: Fig. S4B). The mQTL-SMP associated SNPs were enriched for fewer GO terms, but included pathways such as nuclear chromosome part, response to peptide and chromatin (see Additional file 1: Fig. S4C). However, were only enriched for one KEGG term, AMPK signalling pathway (see Additional file 1: Fig. S4D).

### DNA methylation variation in 5’UTRs at putative epialleles is linked to gene expression variation

**Fig. 5:**
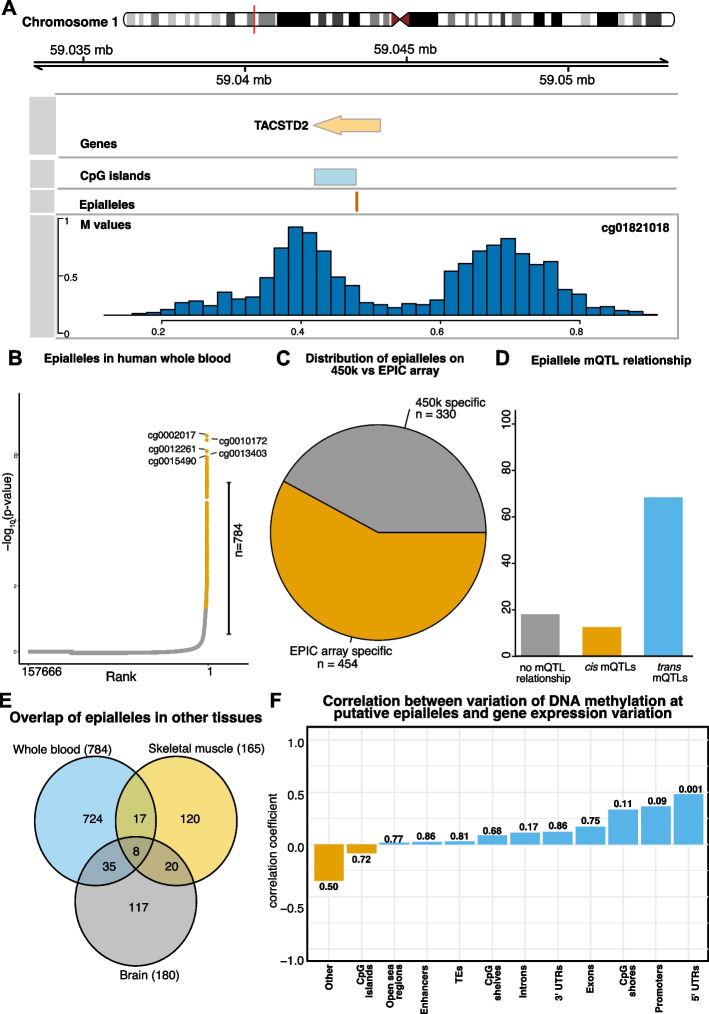
Putative epialleles identified in whole human blood. **A** Genomic plot showing a putative epiallele on chromosome 1 overlapping the TACSTD2 gene. **B** Rank plot showing proportion of CpG sites on the EPIC array which are labelled as epialleles. A total of 784 CpGs met this threshold, and the top 5 are annotated. **C** Pie chart showing distribution of putative epialleles on the Illumina 450k and Illumina EPIC array. **D** Barplot showing percentage of mQTL relationship of putative putative epialleles. Y axis represents percentage. **E** Venn diagram indicating overlap of putative putative epialleles identified in whole blood, brain and skeletal muscle tissue in humans. **F** The correlation between variation in gene expression and variation in methylation of epialleles. The correlation test significance is reported above the bars (*p*-value: n.s. $\ge$ 0.05, *p-value <0.05, ** <0.01 and *** <0.001)

*Epiallele* is a term that has been defined differently and often in negative terms [45], that is as sites which are variably expressed but not alleles, or even more strictly in the absence of a genetic difference [46]. Here, in contrast we screen for *loci* which are present in more than one epigenetic state, which could be stochastic or driven by genetics, environment or parent of origin, all falling under a broad definition of *epiallele*. Some of the variable methylation probes display two peaks, with one subgroup of the population displaying lower methylation at a CpG site and the other subgroup high methylation and only a small percentage of individuals with intermediary methylation values between the two subgroups (Fig. 5A). To investigate this systematically, we screened for putative epialleles in human whole blood by applying a test for unimodality (using the Hartigan’s dip test) in CpG sites which showed an average variable intermediate methylation value between 0.4 and 0.6. A total of 784 CpG sites met this criterion and we labeled them as putative epialleles (see Fig. 5B and Additional file 10). 405 of these putative epialleles are associated with at least one gene, and 52 of these genes contained more than one putative epiallele, with PM20D1 containing 8 putative epiallele (cg05841700, cg07157834, cg07167872, cg07533224, cg12898220, cg14159672, cg16334093 and cg24503407). PM20D1 is a gene which has previously been reported to be associated with obesity, insulin resistance and the progression of Alzheimer’s disease [47, 48]. Moreover, it has previously been reported to contain a variably methylated region associated also with body mass index [49]. Additionally, several genes involved in the major histocompatibility complex such as HLA-DRB1, HLA-DQA1, HLA-C and HLA-DRB6 also contained several CpGs identified as putative epialleles in our analysis.

Of these CpGs, 42% are found on the Illumina 450 K array and 58% were unique to the Illumina EPIC array (see Fig. 5C). Furthermore, we hypothesised that some of these putative epiallele sites may be controlled by genetic variation and found that roughly 19% of these were in fact not linked to any genetic variants, but 81% were. Of these, the majority were *trans* mQTLs (65%) and the others were *cis* mQTLs (16%) (see Fig. 5D).

As we identified that some of these epiallele sites were potentially mediated by genetic variants, we aimed to investigate if these are tissue specific or common between different tissues. Therefore, we calculated these putative epialleles in two other human tissues from different cohorts; 160 skeletal muscle samples [50] and 1, 121 brain samples [51]. We identified limited overlap between all three tissues (Fig. 5E), suggesting that the majority of these putative epialleles are tissue specific. These putative epialleles are also located within tissue specific genes, which further supports the result that they are tissue specific (Additional file 1: Fig. S5A). In addition, 7 of them are located at imprinted loci, including OR2L13 (cg08861456), RGS14 (cg16006841), WDR27 (cg11938672 and cg18322025), BRSK2 (cg08208480), ZNF331 (cg05338009) and GNAS (cg24617313) (Additional file 1: Fig. S5B). The low overlap between epialleles and imprinted regions is not surprising since one expects approximately 50% methylation at imprinted loci in each individual and here we investigate interindividual variation in DNA methylation.

To try to investigate the regulatory role for these epialleles, we also investigated their functional annotations. Interestingly, we find the putative epilleles to be enriched at enhancer and intergenic regions, but depleted at promoters, exonic and 3’UTR regions (Additional file 1: Fig. S5C, D). One possibility is that these epialleles are under control of Polycomb Repressive Complex and, to test that, we considered the co-localisation of epialleles with H3K27me3 (a epigenetic mark deposited by PRC2) for different blood cell lines from ENCODE project [52]. Our analysis showed that only small percentage of epialleles (less than 10%) are located at H3K27me3 ChIP-seq peaks in different blood cell types (Additional file 1: Fig. S5E).

As it has previously been reported that epigenetic variation at epialleles may result in gene expression variation, we next annotated putative epialleles to their nearest target gene in order to investigate this further. We calculated the relationship between methylation variation and target gene expression variation in microarray data using the Pearson’s correlation. Our results show a positive correlation between methylation variation at putative epialleles in 5’UTRs and their target gene expression variation (Fig. 5F). This raises the question whether VMPs in general, rather than putative epialleles, can impact gene expression since they are also enriched at regulatory regions and for TF motifs. We performed the same analysis as for putative epialleles, but in this case we found negligible correlation (Pearson correlation coefficient lower than 0.15) between variability in gene expression and variability in DNA methylation at VMPs, but several are statistically significant (including TEs); Additional file 1: Fig. S6.

To further validate the potential regulatory role of epialleles, we repeated the correlation analysis using matched DNA methylation and gene expression data for 111 individuals available from [53] study. This cohort consisted of approximately 65% females and 35% males and, to include positive controls, we also considered the sex chromosomes expecting that some of the CpGs on X chromosome would be identified as sex associated epialleles. In this smaller dataset, we identified only 6,896 VMPs and 127 epialleles, with 49 epialleles located on the X chromosome. We directly calculated the Pearson correlation between methylation levels and gene expression values across individuals, offering a more direct test of the link between epialleles and gene expression. Similar to the case of the unmatched analysis, we observed a negative correlation between DNA methylation levels of epialleles located in 5’UTR regions and gene expression (Additional file 1: Fig. S7A). When repeating this analysis for all VMPs, we observed a small negative correlation between DNA methylation and gene expression only at enhancers (Additional file 1: Fig. S7B).

Despite the general trend of anti-correlation observed for epialleles in 5’UTR, the strongest negative correlation between DNA methylation and expression was identified at a subset of epialleles located in promoters (cg04863005 at TACSTD2 promoter and cg11717280 and cg20698282 at *Xist* promoter) or TEs (cg16163535 inside MER4CL34 located within an intron of SLC12A7 gene); Additional file 1: Fig. S7C. Identification of epialleles in the promoter of *Xist* (showing two peaks, one at 0.4 and another one at 0.8) is consistent with the activation of *Xist* lncRNA in females samples during X chromosome inactivation [54] and is supported by replotting the data split between males and females (Additional file 1: Fig. S7D). Furthermore, on the analysis of the Understanding Society dataset we identified two epiealleles for TACSTD2 (cg01821018, see Fig. 5 A, and cg04863005), both associated with an mQTL located $100\ Kb$ away (*chr*1 : 58944833) indicating that they are likely under genetic control. Interestingly, the second epieallele was also identified in the dataset from [53] and showed a strong anti-correlation with TACSTD2 expression. Finally, cg16163535 epiallele is also under genetic control with an mQTL located $2\ Kb$ away (chr5:1104938) and while it was identified as a VMP in the Understanding Society dataset, it did not meet the threshold to be classified as an epiallele.

## Discussion

We analysed DNA methylation interindividual variability and stability amongst healthy individuals in a cohort of over 3.6*K* individuals from UK (Understanding Society datasets) in order to better understand its role in diversity in human phenotypes and its relationship with gene expression variation and other genomic factors. The main aim of this study was to quantify interindividual variation in DNA methylation in a large cohort representative for UK population using the EPIC array. When comparing the VMPs discovered in this study to findings previously reported in other studies, we do recover some of the previously reported VMPs, but also identify novel ones (Additional file 1: Fig. S8). For example, in our VMPs, we recover 85.28% of the hyper variable CpGs identified in a previous study in whole blood that are also found in other tissues [17], 43% of previously reported VMPs in different blood cell types from 426 monozygotic twin pairs [14], and 41% of the CpGs from the EPIC array that are located in regions displaying variability in methylation in multiple tissues from [31, 32].

Overall, we recover more CpGs sites that display interindividual variability in DNA methylation (approximately 31 K additional CpGs) and there are two reasons for this. First, our study is the first one to our knowledge that uses the EPIC array to investigate interindividual variation in DNA methylation and approximately half of our VMPs are EPIC array specific (Additional file 1: Fig. S1C). Second, these previous studies investigated CpGs that display interindividual variation in DNA methylation in multiple tissues. When applying our same approach to EPIC data in brain [51] (Additional file 11) and skeletal muscle [50] (Additional file 12), we found that 5,835 VMPs (16.68% of whole blood VMPs) are common between whole blood and brain or skeletal muscle. This is similar to the number of CpGs that display interindividual variation in DNA methylation in multiple tissues in previous studies [17].

Our analysis is rooted in a population that is representative, avoiding biases towards specific disease states or aging. Nevertheless, we identified a relatively small subset of our VMPs that is associated with age-related CpGs. We anticipate that running a similar analysis on datasets curated specifically for aging populations (e.g., [55]) would likely reveal an increased number of VMPs associated with age.

It is important to note that most of population large scale studies are performed in whole blood and, recently, EPIC array become the most used assay for Epigenome Wide Association Studies (70% of EWAS studies deposited on GEO used EPIC array in 2020) as WGBS is prohibitively expensive for large population studies [56]. This is exactly what our study focuses on and having this catalogue of highly variable probes would be of great use when investigating epigenetic signals with EPIC array in whole blood.

Overall, our study indicates that approximately 35 K CpGs vary in a meaningful way on the EPIC array in whole blood. Assaying only those that vary meaningfully could significantly increase the statistical power of an EWAS. However, we do not advocate focusing only on these, since one might miss probes that display variability in certain conditions, disease states or upon exposure to certain environment. Nevertheless, this catalogue of probes can provide additional information when interpreting results from EWAS studies, especially for whole blood.

DNA methylation variability is strongly linked to genomic context and we found that VMPs are enriched in enhancers and intergenic regions which is in contrast to SMPs that are highly enriched in CpG islands and promoter regions. We also found that while SMPs are associated with housekeeping genes, VMPs are more frequently located in tissue-specific regulatory regions and also show significant enrichment at imprinted loci, suggesting a potential role in allele-specific regulation.

Most importantly, in this study, we have performed a systematic characterisation of the VMPs and SMPs to identify potential underlying processes that could explain the interindividual variation in DNA methylation. Consistent with previous research [57], we demonstrated that only a small percentage of these loci overlap regions known to be associated with biological age, biological sex and smoking status. Interestingly, we observed that all of the highly variable sites showed an intermediate methylation status, a finding previously identified by [13]. The role of intermediate methylation remains unclear, however, previous work suggests that it may be a conserved signature of gene regulation and exon usage [58]. Regions of intermediate methylation were also shown to have similar intermediate levels of active chromatin marks and their target genes also having intermediate transcriptional activity. This together with the enrichment of these sites at CpG shores, intergenic regions and enhancers, which was also previously reported in [14], indicate that these sites may have regulatory potential distinct from repressive or permissive states resulting from fully methylated or unmethylated sites. In contrast, the most stable sites show either high or low average DNA methylation values across our cohort, indicating these loci might be under tight epigenetic control. In line with this, we identified an enrichment of SMPs at housekeeping genes, which is in contrast to the enrichment of VMPs at tissue specific genes.

Expanding on this, it has also been reported that highly variable sites are found at imprinted control regions [17]. Through investigation of the genes annotated to our VMPs, we also found 21 VMPs identified in our analysis annotated to the gene HOXA5, a gene predicted to be maternally imprinted. We therefore checked the enrichment of our identified VMPs and SMPs in imprinted regions, and found that the VMPs were significantly enriched in imprinted regions compared to SMPs (permutation test, *p*-value <0.05). This is also in line with previously reported similar results [14, 17, 59].

The relationship between DNA methylation and gene expression is a complex one. For example, it is traditionally thought that DNA methylation at promoter regions is linked directly to transcriptional repression or gene silencing. Despite this, recent work that synthetically methylated thousands of promoters in the human genome failed to identify a link between promoter DNA methylation and gene expression. Yet, they suggest that the context specific roles of DNA methylation are highly influenced by transcription factor binding affinities [60]. Therefore, we investigate the regulatory networks by searching for the presence of transcription factors binding sites among VMPs and SMPs enriched at important regulatory regions such as promoters and enhancers. Interestingly, we identify the presence of transcription factors which have indeed been previously reported to be methylation sensitive. For example, we identify TFAP2A as the most signficantly enriched motif at VMPs at promoters. It has previously been reported that the presence of DNA methylation leads to an increased binding of TFAP2A to B1 TEs, leading to suppressed gene expression of NRBP1 gene [61]. Additionally, we find SREBF1 to be the most significantly enriched TF motif at SMPs at promoters which has also been reported to be sensitive to CpG methylation [62]. Furthermore, our hub analysis for transcription factor motif at VMPs and SMPs located in enhancers and promoters also revealed more methylation sensitive TFs such as JUN, ATF4 BRCA1, FOXA1 and HIF1A [61, 63–65]. This indicates that interindividual differences in DNA methylation would result in differences in TF binding and consequently differences in gene regulation.

It is possible that variance or stability of DNA methylation may also be directed by genetic differences, as previously reported [24]. In line with this, we showed here that VMPs seem to be under higher genetic control than SMPs, suggesting that genetic differences may in part drive epigenetic interindividual variability. It is worthwhile noting that the mQTLs are statistical association analysis that produces thousands of results that can then provide hypotheses for subsequent follow-up functional exploration [66]. We did not exclude mQTL-VMP or mQTL-SMP sites in our analysis, but rather used them to see what percentage of VMPs and SMPs might be directed by genetic differences. Our results showed that approximately half of the VMPs may be directed by genetic differences in contrast with only 3% of SMPs. While there are several ways that genetic differences may result in epigenetic differences in *cis*, the mechanism by which *trans* mQTL-mCpG pairs, identified through mQTL analysis, work in trans is not fully understood. These pairs are crucial for understanding long-range genetic and epigenetic interactions within the genome. We therefore hypothesised that 3D chromatin organisation and DNA methylation are tightly linked based on previous work indicating that TADs play a role in gene regulation [67]. With this aim, we found that majority of *trans* mQTL-VMP pairs were located in the same TAD, but this is not more than we would expect by chance for mQTL-mCpG pairs (see Fig. 3D). This indicates that mQTL pairs (both for VMP mQTLs and non VMP mQTLs) are in part influenced by or influence chromatin organisation, suggesting that 3D genome organisation may in part explain the interindividual variability in DNA methylation (see Fig. 3D). However, it is important to note that although the design of the Illumina EPIC array was curated to include more distal regulatory elements such as enhancers, it is possible that the design of the array was not ideal to answer this research question.

Epialleles are regions at which the epigenetic state varies amongst individuals within a population [68]. Here, we leveraged our catalogue of VMPs as a means of identifying putative epialleles. Using this approach, we were able to identify 784 putative epialleles in human whole blood which showed average intermediate methylation levels across our sample, yet upon closer inspection demonstrated bimodal distribution. We observed that just over half of these are unique to the Illumina EPIC array, meaning we were able to reveal novel putative epialleles. The establishment of these putative epialleles remains unclear, but may result from several factors. One possibility is that the differences in methylation state at these sites is due to genotype. Whilst we screened out CpGs related to SNPs, some epiallele sites may still be influenced by mQTLs. Here, we were able to identify enrichment for mQTLs at putative epialleles and these were mainly *trans* relationships. However, there were still a portion of putative epialleles which were not linked to any SNPs via our mQTL analysis, suggesting that stochastic or environmental exposures may play a role in the establishment of these putative epialleles in whole blood. We further demonstrated that some of these putative epialleles display tissue specificity (Fig. 5E).

We further explored this relationship between DNA methylation variance and target gene expression variance at these putative epialleles and also considered the genomic context in which DNA methylation was found. Interestingly, we observed that DNA methylation variance and target gene expression variance are positively correlated when epialleles are located at 5’UTRs. This identifies a link between DNA methylation at specific regulatory regions and gene regulation. It is important to note, that this analysis was performed in non matching data sets (i.e. the data sets were collected from different cohorts), which further supports the generalisation of these results. More importantly, this link between DNA methylation and gene expression seems to be specific for this putative epialleles, given that a similar analysis over all VMPs was not able to identify similar correlations with gene expression. When analysing a dataset with matched DNA methylation and transcription, we observed a small negative correlation between DNA methylation and gene expression for epialleles located in 5’UTR, but the strongest negative correlation was observed at three epialleles located in promoters and one in a TE. This matched DNA methylation gene expression dataset consisted only of 111 individuals and consequently allowed identification of only 6,896 VMPs and 127 epialleles with approximately 40% of the epialleles on chromosome X. Nevertheless, further experimental validation by targeted epigenetic editing of these epialleles (CRISPRi/a) is required to confirm their potential regulatory function.

It is worthwhile noting that our analysis primarily focuses on patterns of DNA methylation variability in whole blood, which may not fully represent the epigenetic landscape in other tissues or cell types [69]. This highlights the importance of conducting tissue-specific analyses to capture the dynamic nature of the epigenome across different cellular environments. Furthermore, in this study, we did not investigate epigenetic variation at individual cell level, but the methylation levels we are using for each individual are average methylation at the corresponding positions over multiple cells in whole blood. Additionally, despite the improved coverage provided of the Illumina EPIC array, it does not cover the entire epigenome and therefore studies like this may overlook regions of relevance that could contribute to interindividual variability [31]. Moreover, while our study benefits from a large sample size, it may not fully capture the diversity of DNA methylation patterns across various populations or under different environmental conditions [55].

Finally, one mechanism underlying DNA methylation variability that we did not explore in this study is active demethylation mediated by Tet enzymes or the distinction between 5-methylcytosine (5mC) and 5-hydroxymethylcytosine (5hmC), which cannot be differentiated using the Illumina EPIC array. Future research incorporating techniques such as oxidative bisulfite sequencing could provide insights into the variability observed in this study and shed light on the role of 5hmC and active demethylation at VMPs. Additionally, functional experiments and the integration of multi-omic data could provide deeper insights into the mechanisms underlying interindividual epigenetic variation, including its connections to gene expression regulation, genetic influences, and environmental exposures. Overall, our findings disentangle DNA methylation variability and its possible implications for understanding epigenetic regulation and its role in health and disease.

## Conclusions

In this study, we systematically quantified interindividual variability in DNA methylation in a large cohort of 3.6*K* individuals (Understanding Society datasets), which is representative for UK population. DNA methylation was measured using EPIC array and slightly more than half of the VMPs we identified were specific to the EPIC array (not found on 450 K array). Most of these VMPs are in regulatory regions (promoters and enhancers) and are enriched in motifs for TFs including some that show DNA methylation binding sensibility. Furthermore, we showed that half of the VMPs are associated with genetic variation, 15% with cell type composition and approximately one quarter with age, sex and smoking status. Finally, we identified a subset of 784 putative epialleles in human whole blood that show average intermediate methylation levels across our sample and a bimodal distribution, with some of these epialleles linked to changes in gene expression.

## Methods

### Datasets and statistical analysis

In this study, we used whole blood Illumina Infinium MethylationEPIC BeadChip DNA methylation data collected from participants involved in Understanding Society: The UK Household Longitudinal Study; see [70, 71]. We chose these data sets so that we were able to perform our analysis on relatively homogenous populations using a discovery and validation approach. Furthermore, this data was not collected for any various specific exposures such as ageing, smoking behaviour or any health condition, meaning it is a representative dataset for the British population. Thus, the VMPs that we identified in this study are not biased by any exposure or health condition. Raw signal intensities were processed using the R package bigmelon [72] from IDAT files and used same preprocessing steps as in [71]. Briefly, the data was then normalised via the interpolatedXY adjusted dasen method (which specifically eliminates biases between Type I and Type II probes) implemented in the R package, watermelon [73]. Following normalisation of the data, SNP probes, cross hybridizing probes [74] and X or Y linked probes were removed from the data set. Moreover, as sex of our samples was self reported, we also utilised a DNA methylation based sex classifier [75] in order to remove samples where reported and biological sex did not match. This resulted in 4 and 11 samples being removed from our discovery and validation data sets, respectively. Lastly, to avoid the possibility that this analysis would flag up SNPs from our sample, we looked for confounding between SNPs and CpG sites using the MethylToSNP R package [76]. The function *MethylToSNP* extracts CpG sites corresponding to genotypes CT,TT and CC using 3 discrete levels of methylation: fully methylated, fully unmethylated, and 50% methylation. This resulted in the identification of CpG sites confounding with SNPs and these were also removed from our analysis. The final discovery and validation data set consisted of 1, 171 and 2, 471 samples respectively, and 747, 302 DNA methylation sites.

### Identifying variable and stable sites on the Illumina EPIC array

To identify variably methylated and stably methylated probes on the Illumina EPIC array across individuals, we applied a downsampling approach. First, to ensure we detect robust variable and stable sites, in step 1, we downsampled the discovery data set by 10% (by removing randomly 10% of participants in the dataset). This will minimise the risk of spurious classifications driven by outlier individuals or sample specific effects. Second, to measure variability and stability, in step 2, we calculated standard deviation (SD) of methylation values for each probe across all individuals in our downsampled dataset. Those with the top 10% highest SD values were labelled as variable methylated probes (VMPs) and those with the 10% lowest SD values were labelled as the stable methylated probes (SMPs). Note that this is consistent to the approach in [17], where the top 5% variable sites were selected when using the 450 K array. In our study, we used the EPIC array (having twice more probes) and, thus, we selected the top 10% variable or stable sites. We repeated the downsampling (step 1) and identification of VMPs and SMPs (step 2) ten times and kept only the VMPs and SMPs probes that appeared in all ten downsampled datasets. This analysis was then repeated in our validation dataset and only the VMPs and SMPs that were identified in both discovery and validation datasets were carried forward for further analysis. This provided us with a highly robust catalogue of CpG sites which had either variable (VMPs) or stable (SMPs) DNA methylation patterns (see Additional files 2 and 3).

Cell type proportions were calculated with the estimateCellCounts function from the minfi package [77], for all of the subjects. This uses the Houseman method [78] to estimate the proportions of six cell types: CD8T, CD4T, NK, B cells, Monocyte and Granulocyte. To investigate the probewise variability that is not due to cell type variability, we produced a data set that was corrected for cell proportion using a linear model. The estimates for the six cell types sum to nearly 1, indicating that, for this model, we would need to use just 5 of the estimates (since the sixth is the remainder and, thus, not independent). Relative to abundance, the NK estimate has the highest variance. Experiments with probes including the strongly cell-type associated cg12976793 indicated that the residuals of a linear model including all but the NK estimate retained the best correlation of the residual with the original betas, so we applied this model to both data sets. To make a corrected data set more comparable to the original betas for analysis, we used the sum of the intercept (for each probe) with the residuals (for each individual and probe). We then repeated the complete main analysis on this corrected data set.

### Housekeeping and tissue specific gene annotations

We utilised expression data for 57 tissue types from the Epigenome Roadmap [69] and annotated housekeeping genes by assessing those genes where expression was in the top 40th percentile in all 57 samples, as previously described in [67]. All other genes were then annotated as tissue specific genes.

### Genomic annotations and gene ontology analyses

We annotated CpG sites using the manufacturer supplied annotation data (MethylationEPIC_v-1-0_B2 manifest file). Annotation was completed in the R package Minfi [77]. Several categories were used as annotations in relation to CpG islands and divided into the following categories: CGIs, CGI shores (S and N), CGI shelfs (S and N) and open sea regions. Furthermore, we also annotated the autosomal CpGs to several genomic features, including exons, introns, 5’UTR, 3’UTR, enhancers, promoters and transposable elements (TEs) using data from UCSC table browser (https://genome.ucsc.edu/cgi-bin/hgTables). GO analyses were conducted using the gometh function in the missMethyl package [79], which tests gene ontology enrichment for significant CpGs while accounting for biases inherent in the array design (differing number of probes per gene present on the EPIC array). For GO ontology analyses of enriched TFBS we used enrichGO and enrichKEGG from the clusterProfiler package in R [80], which performs false discovery rate (FDR) adjustment.

### Protein-protein network visualisation and hub gene identification

We searched all of the transcription factors enriched at VMPs and SMPs using the Search Tool for the Retrieval of Interacting Genes (STRING) (https:://string-db.org) database to generate our TF networks. We extracted protein-protein interactions with a combined score greater than 0.4. Following this, we utilised the cytoscape plugin tool Cytohubba [81] to characterise hub genes within the transcription factor networks. This was done by employing the local based method called maximum clique centrality (MCC).

### Enrichment of variably methylated probes and stably methylated probes in transcription factor binding motifs

The enrichment analysis of known TF motifs at VMPs and SMPs at promoters and enhancers was performed using the R package PWMEnrich [82] using the MotifDb collection of TF motifs [83]. Specifically, the DNA sequences within a 50 bp range from the VMPs were extracted from the genome and compared to the SMPs as the background to reveal unique enriched motifs (adjusted *p*-value smaller than 0.05) and vice versa. Using the SMPs as background for TF motifs enriched at VMPs and vice-versa accounts for biases in the EPIC array probe locations in different regions of the genome.

### Annotation of VMPs and SMPs to methylation quantitative trait loci

To investigate the proportion of VMPs and SMPs under genetic control, we annotated them to mQTLs using data from a previous study [24]. We further characterised these into *cis*, *trans* and *cis* and *trans* mQTLs. We defined *cis* mQTLs as cases where the SNP and CpG site were located within 500 bp of each other and *trans* as cases where the SNP and the CpG site were greater 500 bp apart. Several CpG sites were annotated to more than one SNP, where one was a *cis* mQTL and another was a *trans* mQTL, thereby we classified these as *cis* and *trans* mQTLs. To annotate the mQTLs SNPs to biological processes we performed KEGG and GO enrichment analyses as previously described (see Genomic annotations and gene ontology analyses).

### Chromatin loops and topologically associated domains

We examined whether the mQTL pairs occupied the same TAD or were connected by loops using Hi-C data available from the GEO under accession number (GSE124974) for white blood cells and neutrophils. Pre-processing of the data was performed using Juicer command line tools [77]. Reads were aligned to the human (hg38) genome using BWA-mem [80] and then pre-processed using the Juicer pre-processing pipeline. We called chromatin loops using the HICCUPS tool from Juicer using a 10 Kb resolution. We also analysed whether our SNP and CpG pairs annotated by the mQTL analysis occupied the same topologically associated domain by using the hicFindTADs tool from HiCExplorer [84]. These steps then allowed us to calculate the percentage of mQTL pairs which were connected via loops or occupying the same TAD compared to the EPIC array background. Lastly, Hi-C maps were generated using the hicPlotTADs function from HiCExplorer.

### Characterising putative epialleles

In order to characterise putative epialleles in human whole blood, we first identified those VMPs which had an average intermediate methylation across our sample as characterised by an average beta value between 0.40 and 0.60. Majority of VMPs display average beta value inside this interval and using this filtering only removes a small percentage of putative epialleles. Following this, we employed Hartigan’s dip test [85] using the diptest package in R to test for unimodality across these sites. We characterised epialleles as sites which had variable average intermediate methylation and bimodal distribution (Hartigan’s dip test, p value smaller than 0.05); see Additional files 8, 13 and 14.

### Integration with gene expression

We investigated the correlation between methylation variation and target gene expression variation at putative epialleles using gene expression microarray data obtained from the study of health in Pomerania (SHIP-Trend). This study is a longitudinal population based study based in Germany, aiming to assess common diseases and their relevant risk factors. Examinations took place from 2008-2012 and originally involved 4, 420 participants. Gene expression levels for a subset of these participants were measured using the Illumina HumanHT-12 v3 BeadChip array from whole blood cells (*n*=991). Details of the pre-processing methods have been previously reported in [86]. We calculated the coefficient of variation (standard deviation of expression value divided by its average expression value) for each gene as a measure of variance. Following this, we then calculated Pearson’s correlation between methylation variation at each putative epiallele site and gene expression variation of their target gene.

We also consider the matched DNA methylation gene expression data in CD14+ monocytes from 111 patients [53]. The methylation data was measured using EPIC array and was preprocessed as above, while the gene expression data was measured by RNA-seq and the already preprocessed tables were used (GSE201754). We calculated the Pearson correlation coefficients between methylation levels at putative epialleles (127) and VMPs (6,896) and the expression levels of their target genes across samples (see Additional files 15 and 16).

## Supplementary information

Additional file 1. Supplementary Figures.

Additional file 2. List of all VMPs in whole blood.

Additional file 3. List of all SMPs in whole blood.

Additional file 4. List of all VMPs in whole blood with low ICC.

Additional file 5. List of all SMPs in whole blood with low ICC.

Additional file 6. List of all VMPs in whole blood that are controlled by cell type composition.

Additional file 7. List of all SMPs in whole blood that are controlled by cell type composition.

Additional file 8. List of all mQTL-VMP pairs.

Additional file 9. List of all mQTL-SMP pairs.

Additional file 10. List of all putative epialleles in whole blood.

Additional file 11. List of all VMPs in brain.

Additional file 12. List of all VMPs in skeletal muscle.

Additional file 13. List of all putative epialleles in brain.

Additional file 14. List of all putative epialleles in skeletal muscle.

Additional file 15. List of all VMPs in CD14+ cells.

Additional file 16. List of all epialleles in CD14+ cells.

## Data Availability

All EPIC data used in this study is available from the UKDA [87]. Customised scripts have been deposited at Zenodo [88] under the GPL3.0 license and on GitHub [89].

## References

[1] Bell JI. Single nucleotide polymorphisms and disease gene mapping. Arthritis Res Ther. 2002;4:1–6. 10.1186/ar555 PMC3240131 12110147

[2] Robert F, Pelletier J. Exploring the impact of single-nucleotide polymorphisms on translation. Front Genet. 2018;9:507. 30425729 10.3389/fgene.2018.00507 PMC6218417

[3] Wong AH, Gottesman II, Petronis A. Phenotypic differences in genetically identical organisms: the epigenetic perspective. Hum Mol Genet. 2005;14(suppl 1):11–8. 10.1093/hmg/ddi116 15809262

[4] Young PE, Kum Jew S, Buckland ME, Pamphlett R, Suter CM. Epigenetic differences between monozygotic twins discordant for amyotrophic lateral sclerosis (ALS) provide clues to disease pathogenesis. PLoS One. 2017;12(8):0182638. 10.1371/journal.pone.0182638 PMC5552194 28797086

[5] Gervin K, Vigeland MD, Mattingsdal M, Hammero M, Nygård H, Olsen AO, et al. Dna methylation and gene expression changes in monozygotic twins discordant for psoriasis: identification of epigenetically dysregulated genes. PLoS Genet. 2012;8(1):1002454. 10.1371/journal.pgen.1002454 PMC3262011 22291603

[6] Vogt J, Kohlhase J, Morlot S, Kluwe L, Mautner V-F, Cooper DN, et al. Monozygotic twins discordant for neurofibromatosis type 1 due to a postzygotic nf1 gene mutation. Hum Mutat. 2011;32(6):2134–47. 10.1002/humu.21476 21618341

[7] Millan-Zambrano G, Burton A, Bannister AJ, Schneider R. Histone post-translational modifications—cause and consequence of genome function. Nat Rev Genet. 2022;23:563–80. 10.1038/s41576-022-00468-7. 35338361 10.1038/s41576-022-00468-7

[8] Statello L, Guo C-J, Chen L-L, Huarte M. Gene regulation by long noncoding rnas and its biological functions. Nat Rev Mol Cell Biol. 2021;22:96–118. 10.1038/s41580-020-00315-9. 33353982 10.1038/s41580-020-00315-9 PMC7754182

[9] Gibney ER, Nolan CM. Epigenetics and gene expression. Heredity. 2010;105:4–13. 10.1038/hdy.2010.54. 20461105 10.1038/hdy.2010.54

[10] Greenberg MVC, Bourc’his D. The Diverse Roles of DNA Methylation in Mammalian Development and Disease. 10.1038/s41580-019-0159-6. 10.1038/s41580-019-0159-6 31399642

[11] Heyn H, Moran S, Hernando-Herraez I, Sayols S, Gomez A, Sandoval J, et al. Dna methylation contributes to natural human variation. Genome Res. 2013;23:1363–72. 10.1101/gr.154187.112. 23908385 10.1101/gr.154187.112 PMC3759714

[12] Bock C, Walter J, Paulsen M, Lengauer T. Inter-individual variation of *dna* methylation and its implications for large-scale epigenome mapping. Nucleic Acids Res. 2008;36(10):55. 10.1093/nar/gkn122 PMC2425484 18413340

[13] Hachiya T, Furukawa R, Shiwa Y, Ohmomo H, Ono K, Katsuoka F, et al. Genome-wide identification of inter-individually variable *dna* methylation sites improves the efficacy of epigenetic association studies. NPJ Genom Med. 2017;2(1):1–14. 29263827 10.1038/s41525-017-0016-5 PMC5677974

[14] Garg P, Joshi RS, Watson C, Sharp AJ. A survey of inter-individual variation in *dna* methylation identifies environmentally responsive co-regulated networks of epigenetic variation in the human genome. PLoS Genet. 2018;14(10):1007707. 10.1371/journal.pgen.1007707 PMC6181428 30273333

[15] Costello KR, Leung A, Trac C, Lee M, Basam M, Pospisilik JA, Schones DE. Sequence features of retrotransposons allow for epigenetic variability. Elife. 2021;10. 10.7554/eLife.71104 PMC8555987 34668484

[16] Palumbo D, Affinito O, Monticelli A, Cocozza S. *Dna* methylation variability among individuals is related to cpgs cluster density and evolutionary signatures. BMC Genomics. 2018;19(1):1–9. 29606093 10.1186/s12864-018-4618-9 PMC5880022

[17] Derakhshan M, Kessler NJ, Ishida M, Demetriou C, Brucato N, Moore GE, et al. Tissueand ethnicity-independent hypervariable DNA methylation states show evidence of establishment in the early human embryo. Nucleic Acids Res. 2022;50(12):6735–52. 35713545 10.1093/nar/gkac503 PMC9749461

[18] Rakyan VK, Hildmann T, Novik KL, Lewin J, Tost J, Cox AV, et al. DNA methylation profiling of the human major histocompatibility complex: a pilot study for the human epigenome project. PLoS Biol. 2004;2(12):405. 10.1371/journal.pbio.0020405 PMC529316 15550986

[19] Schröder C, Leitão E, Wallner S, Schmitz G, Klein-Hitpass L, Sinha A, et al. Regions of common inter-individual dna methylation differences in human monocytes: genetic basis and potential function. Epigenetics Chromatin. 2017;10(1):1–18. 28747224 10.1186/s13072-017-0144-2 PMC5530492

[20] Flanagan JM, Popendikyte V, Pozdniakovaite N, Sobolev M, Assadzadeh A, Schumacher A, et al. Intra-and interindividual epigenetic variation in human germ cells. The American Journal of Human Genetics. 2006;79(1):67–84. 16773567 10.1086/504729 PMC1474120

[21] El-Maarri O, Kareta MS, Mikeska T, Becker T, Diaz-Lacava A, Junen J, et al. A systematic search for dna methyltransferase polymorphisms reveals a rare dnmt3l variant associated with subtelomeric hypomethylation. Hum Mol Genet. 2009;18(10):1755–68. 19246518 10.1093/hmg/ddp088

[22] Hwang DG, Green P. Bayesian markov chain monte carlo sequence analysis reveals varying neutral substitution patterns in mammalian evolution. Proc Natl Acad Sci U S A. 2004;101:13994–4001. 10.1073/pnas.0404142101. 15292512 10.1073/pnas.0404142101 PMC521089

[23] Gao X, Thomsen H, Zhang Y, Breitling LP, Brenner H. The impact of methylation quantitative trait loci (mqtls) on active smoking-related dna methylation changes. Clin Epigenetics. 2017;9(1):1–13. 28824732 10.1186/s13148-017-0387-6 PMC5561570

[24] Hannon E, Gorrie-Stone TJ, Smart MC, Burrage J, Hughes A, Bao Y, et al. Leveraging dna-methylation quantitativetrait loci to characterize the relationship between methylomic variation, gene expression, and complex traits. The American Journal of Human Genetics. 2018;103(5):654–65. 30401456 10.1016/j.ajhg.2018.09.007 PMC6217758

[25] Hannon E, Spiers H, Viana J, Pidsley R, Burrage J, Murphy TM, Troakes C, Turecki G, O’donovan MC, Schalkwyk LC, et al. Methylation qtls in the developing brain and their enrichment in schizophrenia risk loci. Nat Neurosci. 2016;19(1):48–54. 10.1038/nn.4182 PMC4714325 26619357

[26] Gibbs JR, Van Der Brug MP, Hernandez DG, Traynor BJ, Nalls MA, Lai S-L, et al. Abundant quantitative trait loci exist for dna methylation and gene expression in human brain. PLoS Genet. 2010;6(5):1000952. 10.1371/journal.pgen.1000952 PMC2869317 20485568

[27] Drong AW, Nicholson G, Hedman ÅK, Meduri E, Grundberg E, Small KS, et al. The presence of methylation quantitative trait loci indicates a direct genetic influence on the level of dna methylation in adipose tissue. PLoS ONE. 2013;8(2):55923. 10.1371/journal.pone.0055923 PMC3576415 23431366

[28] Gaunt TR, Shihab HA, Hemani G, Min JL, Woodward G, Lyttleton O, et al. Systematic identification of genetic influences on methylation across the human life course. Genome Biol. 2016;17(1):1–14. 27036880 10.1186/s13059-016-0926-z PMC4818469

[29] Olsson AH, Volkov P, Bacos K, Dayeh T, Hall E, Nilsson EA, et al. Genome-wide associations between genetic and epigenetic variation influence mrna expression and insulin secretion in human pancreatic islets. PLoS Genet. 2014;10(11):1004735. 10.1371/journal.pgen.1004735 PMC4222689 25375650

[30] Bao Y, Gorrie-Stone T, Hannon E, Hughes A, Andrayas A, Neilson G, et al. Social mobility across the lifecourse and dna methylation age acceleration in adults in the uk. Sci Rep. 2022;12(1):1–12. 36566336 10.1038/s41598-022-26433-2 PMC9790005

[31] Gunasekara CJ, Scott CA, Laritsky E, Baker MS, MacKay H, Duryea JD, et al. A genomic atlas of systemic interindividual epigenetic variation in humans. Genome Biol. 2019;20:1–12. 10.1186/s13059-019-1708-1. 31155008 10.1186/s13059-019-1708-1 PMC6545702

[32] Gunasekara CJ, MacKay H, Scott CA, Li S, Laritsky E, Baker MS, et al. Systemic interindividual epigenetic variation in humans is associated with transposable elements and under strong genetic control. Genome Biol. 2023;24:2. 10.1186/s13059-022-02827-3. 36631879 10.1186/s13059-022-02827-3 PMC9835319

[33] Butcher LM, Ito M, Brimpari M, Morris TJ, Soares FAC, ahrlund-Richter L, et al. Non-cg dna methylation is a biomarker for assessing endodermal differentiation capacity in pluripotent stem cells. Nat Commun. 2016;7:10458. 10.1038/ncomms10458. 26822956 10.1038/ncomms10458 PMC4740175

[34] Sugden K, Hannon EJ, Arseneault L, Belsky DW, Corcoran DL, Fisher HL, et al. Patterns of reliability: assessing the reproducibility and integrity of dna methylation measurement. Patterns. 2020;1:100014. 10.1016/j.patter.2020.100014. 32885222 10.1016/j.patter.2020.100014 PMC7467214

[35] Xu Z, Taylor JA. Reliability of DNA methylation measures using illumina methylation beadchip. Epigenetics. 2021;16:495–502. 10.1080/15592294.2020.1805692. 32749174 10.1080/15592294.2020.1805692 PMC8078668

[36] Zhang W, Young JI, Gomez L, Schmidt MA, Lukacsovich D, Varma A, et al. Critical evaluation of the reliability of DNA methylation probes on the illumina methylationepic v1.0 beadchip microarrays. Epigenetics. 2024;19(1):2333660. 10.1080/15592294.2024.2333660. 38564759 10.1080/15592294.2024.2333660 PMC10989698

[37] Salas LA, Koestler DC, Butler RA, Hansen HM, Wiencke JK, Kelsey KT, et al. An optimized library for reference-based deconvolution of whole-blood biospecimens assayed using the illumina humanmethylationepic beadarray. Genome Biol. 2018;19:64. 10.1186/s13059-018-1448-7. 29843789 10.1186/s13059-018-1448-7 PMC5975716

[38] Pervjakova N, Kasela S, Morris AP, Kals M, Metspalu A, Lindgren CM, et al. Imprinted genes and imprinting control regions show predominant intermediate methylation in adult somatic tissues. Epigenomics. 2016;8(6):789–99. 27004446 10.2217/epi.16.8 PMC5066126

[39] Yin Y, Morgunova E, Jolma A, Kaasinen E, Sahu B, Khund-Sayeed S, et al. Impact of cytosine methylation on DNA binding specificities of human transcription factors. Science. 2017. 10.1126/SCIENCE.AAJ2239. 28473536 10.1126/science.aaj2239 PMC8009048

[40] Kribelbauer JF, Laptenko O, Chen S, Martini GD, Freed-Pastor WA, Prives C, et al. Quantitative analysis of the DNA methylation sensitivity of transcription factor complexes. Cell Rep. 2017;19(11):2383–95. 10.1016/j.celrep.2017.05.069. 28614722 10.1016/j.celrep.2017.05.069 PMC5533174

[41] Ravichandran M, Rafalski D, Davies CI, Ortega-Recalde O, Nan X, Glanfield CR, et al. Pronounced sequence specificity of the tet enzyme catalytic domain guides its cellular function. Sci Adv. 2022;8:2427. 10.1126/SCIADV.ABM2427. 10.1126/sciadv.abm2427 PMC9451156 36070377

[42] Hannon E, Gorrie-Stone TJ, Smart MC, Burrage J, Hughes A, Bao Y, et al. Leveraging DNA-methylation quantitativetrait loci to characterize the relationship between methylomic variation, gene expression, and complex traits. Am J Hum Genet. 2018;103:654–65. 10.1016/j.ajhg.2018.09.007. 30401456 10.1016/j.ajhg.2018.09.007 PMC6217758

[43] Peng Q, Liu X, Li W, Jing H, Li J, Gao X, et al. Analysis of blood methylation quantitative trait loci in east Asians reveals ancestry-specific impacts on complex traits. Nat Genet. 2024;56:846–60. 10.1038/s41588-023-01494-9. 38641644 10.1038/s41588-023-01494-9

[44] Ramírez F, Bhardwaj V, Arrigoni L, Lam KC, Grüning BA, Villaveces J, et al. High-resolution tads reveal dna sequences underlying genome organization in flies. Nat Commun. 2018;9(1):1–15. 29335486 10.1038/s41467-017-02525-w PMC5768762

[45] Le Goff A, Allard P, Landecker H. Heritable changeability: epimutation and the legacy of negative definition in epigenetic concepts. Stud Hist Philos Sci. 2021;86:35–46. 10.1016/j.shpsa.2020.12.006. 33965662 10.1016/j.shpsa.2020.12.006

[46] Dolinoy DC, Das R, Weidman JR, Jirtle RL. Metastable epialleles, imprinting, and the fetal origins of adult diseases. Pediatr Res. 2007;61(7):30–7. 10.1203/pdr.0b013e31804575f7 17413847

[47] Wang Q, Chen Y, Readhead B, Chen K, Su Y, Reiman EM, et al. Longitudinal data in peripheral blood confirm that pm20d1 is a quantitative trait locus (qtl) for alzheimer’s disease and implicate its dynamic role in disease progression. Clin Epigenetics. 2020;12(1):1–18. 10.1186/s13148-020-00984-5 PMC7724832 33298155

[48] Yang R, Hu Y, Lee CH, Liu Y, Diaz-Canestro C, Fong CHY, et al. PM20D1 is a circulating biomarker closely associated with obesity, insulin resistance and metabolic syndrome. Eur J Endocrinol. 2022;186(2):151–61. 10.1530/EJE-21-0847 34757919

[49] Feinberg AP, Irizarry RA, Fradin D, Aryee MJ, Murakami P, Aspelund T, et al. Personalized epigenomic signatures that are stable over time and covary with body mass index. Science translational medicine. 2010;2(49):49–674967. 10.1126/scitranslmed.3001262 PMC3137242 20844285

[50] Voisin S, Harvey NR, Haupt LM, Griffiths LR, Ashton KJ, Coffey VG, et al. An epigenetic clock for human skeletal muscle. J Cachexia Sarcopenia Muscle. 2020;11(4):887–98. 32067420 10.1002/jcsm.12556 PMC7432573

[51] Shireby G, Dempster E, Policicchio S, Smith R, Pishva E, Chioza B, Davies J, Burrage J, Lunnon K, Seiler-Vellame D, et al. Dna methylation signatures of alzheimer’s disease neuropathology in the cortex are primarily driven by variation in non-neuronal cell-types. bioRxiv. 2022. 10.1038/s41467-022-33394-7 PMC9509387 36153390

[52] Sloan CA, Chan ET, Davidson JM, Malladi VS, Strattan JS, Hitz BC, et al. Encode data at the encode portal. Nucleic Acids Res. 2016;44:726–32. 10.1093/NAR/GKV1160. 10.1093/nar/gkv1160 PMC4702836 26527727

[53] Estupinan-Moreno E, Ortiz-Fernandez L, Li T, Hernandez-Rodriguez J, Ciudad L, Andres-Leon E, et al. Methylome and transcriptome profiling of giant cell arteritis monocytes reveals novel pathways involved in disease pathogenesis and molecular response to glucocorticoids. Ann Rheum Dis. 2022;81:1290–300. 10.1136/annrheumdis-2022-222156. 35705375 10.1136/annrheumdis-2022-222156 PMC9380516

[54] Loda A, Heard E. Xist RNA in action: past, present, and future. PLoS Genet. 2019;15:1008333. 10.1371/JOURNAL.PGEN.1008333. 10.1371/journal.pgen.1008333 PMC6752956 31537017

[55] Seeboth A, McCartney DL, Wang Y, Hillary RF, Stevenson AJ, Walker RM, et al. DNA methylation outlier burden, health, and ageing in generation scotland and the lothian birth cohorts of 1921 and 1936. Clin Epigenetics. 2020;12:49. 10.1186/s13148-020-00838-0. 32216821 10.1186/s13148-020-00838-0 PMC7098133

[56] Campagna MP, Xavier A, Lechner-Scott J, Maltby V, Scott RJ, Butzkueven H, et al. Epigenome-wide association studies: current knowledge, strategies and recommendations. Clin Epigenetics. 2021;13:214. 10.1186/s13148-021-01200-8. 34863305 10.1186/s13148-021-01200-8 PMC8645110

[57] Lam LL, Emberly E, Fraser HB, Neumann SM, Chen E, Miller GE, et al. Factors underlying variable dna methylation in a human community cohort. Proc Natl Acad Sci. 2012;109(supplement 2):17253–60. 23045638 10.1073/pnas.1121249109 PMC3477380

[58] Elliott G, Hong C, Xing X, Zhou X, Li D, Coarfa C, et al. Intermediate DNA methylation is a conserved signature of genome regulation. Nat Commun. 2015;6(1):1–10. 10.1038/ncomms7363 PMC4333717 25691127

[59] Zeng Y, Amador C, Xia C, Marioni R, Sproul D, Walker RM, et al. Parent of origin genetic effects on methylation in humans are common and influence complex trait variation. Nat Commun. 2019;10(1):1–13. 30918249 10.1038/s41467-019-09301-y PMC6437195

[60] Mendoza A, Nguyen TV, Ford E, Poppe D, Buckberry S, Pflueger J, et al. Large-scale manipulation of promoter DNA methylation reveals context-specific transcriptional responses and stability. Genome Biol. 2022;23(1):1–31. 35883107 10.1186/s13059-022-02728-5 PMC9316731

[61] Zhu Z, Meng W, Liu P, Zhu X, Liu Y, Zou H. DNA hypomethylation of a transcription factor binding site within the promoter of a gout risk gene nrbp1 upregulates its expression by inhibition of tfap2a binding. Clin Epigenetics. 2017;9(1):1–9. 28932319 10.1186/s13148-017-0401-z PMC5603049

[62] Krause C, Geisler C, Tackenberg H, El Gammal AT, Wolter S, Spranger J, et al. Multi-layered epigenetic regulation of irs2 expression in the liver of obese individuals with type 2 diabetes. Diabetologia. 2020;63(10):2182–93. 32710190 10.1007/s00125-020-05212-6 PMC7476982

[63] Luo X, Zhang T, Zhai Y, Wang F, Zhang S, Wang G. Effects of DNA methylation on tfs in human embryonic stem cells. Front Genet. 2021;12:639461. 33708244 10.3389/fgene.2021.639461 PMC7940757

[64] Héberlé É, Bardet AF. Sensitivity of transcription factors to dna methylation. Essays Biochem. 2019;63(6):727–41. 31755929 10.1042/EBC20190033 PMC6923324

[65] D’Anna F, Van Dyck L, Xiong J, Zhao H, Berrens RV, Qian J, et al. DNA methylation repels binding of hypoxia-inducible transcription factors to maintain tumor immunotolerance. Genome Biol. 2020. 10.1186/s13059-020-02087-z. 32718321 10.1186/s13059-020-02087-z PMC7384226

[66] Min JL, Hemani G, Hannon E, Dekkers KF, Castillo-Fernandez J, Luijk R, Carnero-Montoro E, Lawson DJ, Burrows K, Suderman M, Bretherick AD, Richardson TG, Klughammer J, Iotchkova V, Sharp G, Khleifat AA, Shatunov A, Iacoangeli A, McArdle WL, Ho KM, Kumar A, Soderhall C, Soriano-Tarraga C, Giralt-Steinhauer E, Kazmi N, Mason D, McRae AF, Corcoran DL, Sugden K, Kasela S, Cardona A, Day FR, Cugliari G, Viberti C, Guarrera S, Lerro M, Gupta R, Bollepalli S, Mandaviya P, Zeng Y, Clarke TK, Walker RM, Schmoll V, Czamara D, Ruiz-Arenas C, Rezwan FI, Marioni RE, Lin T, Awaloff Y, Germain M, Aissi D, Zwamborn R, Eijk K, Dekker A, Dongen J, Hottenga JJ, Willemsen G, Xu CJ, Barturen G, Catala-Moll F, Kerick M, Wang C, Melton P, Elliott HR, Shin J, Bernard M, Yet I, Smart M, Gorrie-Stone T, Shaw C, Chalabi AA, Ring SM, Pershagen G, Melen E, Jimenez-Conde J, Roquer J, Lawlor DA, Wright J, Martin NG, Montgomery GW, Moffitt TE, Poulton R, Esko T, Milani L, Metspalu A, Perry JRB, Ong KK, Wareham NJ, Matullo G, Sacerdote C, Panico S, Caspi A, Arseneault L, Gagnon F, Ollikainen M, Kaprio J, Felix JF, Rivadeneira F, Tiemeier H, IJzendoorn MH, Uitterlinden AG, Jaddoe VWV, Haley C, McIntosh AM, Evans KL, Murray A, Raikkonen K, Lahti J, Nohr EA, Sorensen TIA, Hansen T, Morgen CS, Binder EB, Lucae S, Gonzalez JR, Bustamante M, Sunyer J, Holloway JW, Karmaus W, Zhang H, Deary IJ, Wray NR, Starr JM, Beekman M, Heemst D, Slagboom, PE, Morange PE, Tregouet DA, Veldink JH, Davies GE, Geus EJC, Boomsma DI, Vonk JM, Brunekreef B, Koppelman GH, Alarcon-Riquelme ME, Huang RC, Pennell CE, Meurs J, Ikram MA, Hughes AD, Tillin T, Chaturvedi N, Pausova Z, Paus T, Spector TD, Kumari M, Schalkwyk LC, Visscher PM, Smith GD, Bock C, Gaunt TR, Bell JT, Heijmans BT, Mill J, Relton CL. Genomic and phenotypic insights from an atlas of genetic effects on dna methylation. Nat Genet. 2021;53:1311–1321. https://doi.org/10.1038/S41588-021-00923-X;TECHMETA=43,45;SUBJMETA=176,2056,208,308,631,692;KWRD=EPIGENETICS,GENETICS+RESEARCH. 10.1038/s41588-021-00923-x PMC7612069 34493871

[67] Chathoth KT, Mikheeva LA, Crevel G, Wolfe JC, Hunter I, Beckett- Doyle S, et al. The role of insulators and transcription in 3d chromatin organization of flies. Genome Res. 2022;32(4):682–98. 35354608 10.1101/gr.275809.121 PMC8997359

[68] Finer S, Holland ML, Nanty L, Rakyan VK. The hunt for the epiallele. Environ Mol Mutagen. 2011;52(1):1–11. 20839222 10.1002/em.20590

[69] Bernstein BE, Stamatoyannopoulos JA, Costello JF, Ren B, Milosavljevic A, Meissner A, et al. The NIH roadmap epigenomics mapping consortium. Nat Biotechnol. 2010;28(10):1045–8. 20944595 10.1038/nbt1010-1045 PMC3607281

[70] Hughes A, Smart M, Gorrie-Stone T, Hannon E, Mill J, Bao Y, et al. Socioeconomic position and DNA methylation age acceleration across the life course. Am J Epidemiol. 2018;187:2346–54. 10.1093/aje/kwy155. 30060108 10.1093/aje/kwy155 PMC6211240

[71] Grant OA, Wang Y, Kumari M, Zabet NR, Schalkwyk L. Characterising sex differences of autosomal DNA methylation in whole blood using the illumina epic array. Clin Epigenetics. 2022;14(1):1–16. 35568878 10.1186/s13148-022-01279-7 PMC9107695

[72] Gorrie-Stone TJ. Dna methylation: Methods and analyses. 2019.

[73] Pidsley R, Wong CC, Volta M, Lunnon K, Mill J, Schalkwyk LC. A data-driven approach to preprocessing illumina 450k methylation array data. BMC Genomics. 2013;14(1):1–10. 23631413 10.1186/1471-2164-14-293 PMC3769145

[74] Chen YA, Lemire M, Choufani S, Butcher DT, Grafodatskaya D, Zanke BW, et al. Discovery of cross-reactive probes and polymorphic cpgs in the illumina infinium humanmethylation450 microarray. Epigenetics. 2013;8:203–9. 10.4161/epi.23470. 23314698 10.4161/epi.23470 PMC3592906

[75] Wang Y, Hannon E, Grant OA, Gorrie-Stone TJ, Kumari M, Mill J, et al. DNA methylation-based sex classifier to predict sex and identify sex chromosome aneuploidy. BMC Genomics. 2021;22(1):1–11. 34182928 10.1186/s12864-021-07675-2 PMC8240370

[76] LaBarre BA, Goncearenco A, Petrykowska HM, Jaratlerdsiri W, Bornman M, Hayes VM, et al. Methyltosnp: identifying snps in illumina DNA methylation array data. Epigenetics Chromatin. 2019;12(1):1–14. 31861999 10.1186/s13072-019-0321-6 PMC6923858

[77] Aryee MJ, Jaffe AE, Corrada-Bravo H, Ladd-Acosta C, Feinberg AP, Hansen KD, et al. Minfi: a flexible and comprehensive bioconductor package for the analysis of infinium DNA methylation microarrays. Bioinformatics. 2014;30:1363–9. 10.1093/bioinformatics/btu049. 24478339 10.1093/bioinformatics/btu049 PMC4016708

[78] Houseman EA, Accomando WP, Koestler DC, Christensen BC, Marsit CJ, Nelson HH, et al. Dna methylation arrays as surrogate measures of cell mixture distribution. BMC Bioinformatics. 2012. 10.1186/1471-2105-13-86. 22568884 10.1186/1471-2105-13-86 PMC3532182

[79] Phipson B, Maksimovic J, Oshlack A. Missmethyl: an R package for analyzing data from illumina?s humanmethylation450 platform. Bioinformatics. 2016;32(2):286–8. 26424855 10.1093/bioinformatics/btv560

[80] Yu G, Wang LG, Han Y, He QY. Clusterprofiler: an R package for comparing biological themes among gene clusters. OMICS. 2012;16:284–7. 10.1089/omi.2011.0118. 22455463 10.1089/omi.2011.0118 PMC3339379

[81] Chin C-H, Chen S-H, Wu H-H, Ho C-W, Ko M-T, Lin C-Y. Cytohubba: identifying hub objects and sub-networks from complex interactome. BMC Syst Biol. 2014. 10.1186/1752-0509-8-S4-S11. 25521941 10.1186/1752-0509-8-S4-S11 PMC4290687

[82] R DDS. Pwmenrich: Pwm enrichment analysis. R package verion 4.26.0. 2020.

[83] Shannon P, Markiel A, Ozier O, Baliga NS, Wang JT, Ramage D, et al. Cytoscape: a software environment for integrated models of biomolecular interaction networks. Genome Res. 2003;13:2498–504. 10.1101/gr.1239303. 14597658 10.1101/gr.1239303 PMC403769

[84] Wolff J, Backofen R, Grüning B. Loop detection using hi-c data with hicexplorer. Gigascience. 2022. 10.1093/gigascience/giac061. 35809047 10.1093/gigascience/giac061 PMC9270730

[85] Hartigan JA, Hartigan PM. The dip test of unimodality. Ann Stat. 1985. 10.1214/aos/1176346577.

[86] Schurmann C, Heim K, Schillert A, Blankenberg S, Carstensen M, Dörr M, et al. Analyzing illumina gene expression microarray data from different tissues: methodological aspects of data analysis in the metaxpress consortium. PLoS One. 2012;7(12):50938. 10.1371/journal.pone.0050938 PMC3517598 23236413

[87] Institute for Social and Economic Research. University of Essex: Understanding Society: Waves 2–3 Nurse Health Assessment, 2010–2012. [data collection]. 6th ed. UK. The European Genome-phenome Archive. 2018. https://ega-archive.org/studies/EGAS00001002836.

[88] Grant OA, Kumari M, Schalkwyk L, Zabet NR. Systematic investigation of interindividual variation of DNA methylation in human whole blood. Zenodo. 2026. 10.5281/zenodo.18542899. 10.1186/s13059-026-04021-1 PMC13069719 41781971

[89] Grant OA, Kumari M, Schalkwyk L, Zabet NR. Systematic investigation of interindividual variation of DNA methylation in human whole blood. GitHub. 2026. https://github.com/livygrant97/interindividualVariation. 10.1186/s13059-026-04021-1 PMC13069719 41781971

