# Supplementary material for: Systematic investigation of interindividual variation of DNA methylation in human whole blood
Source: Genome Biol. 2026 Mar 5;27:128. doi: 10.1186/s13059-026-04021-1 (PMC13069719; doi:10.1186/s13059-026-04021-1)
Supplement: Supplementary file 1 — Additional file 1. Supplementary Figures. [file 13059_2026_4021_MOESM1_ESM.pdf]

Supplementary Material to:
Systematic investigation of interindividual variation of DNA methylation in human whole blood

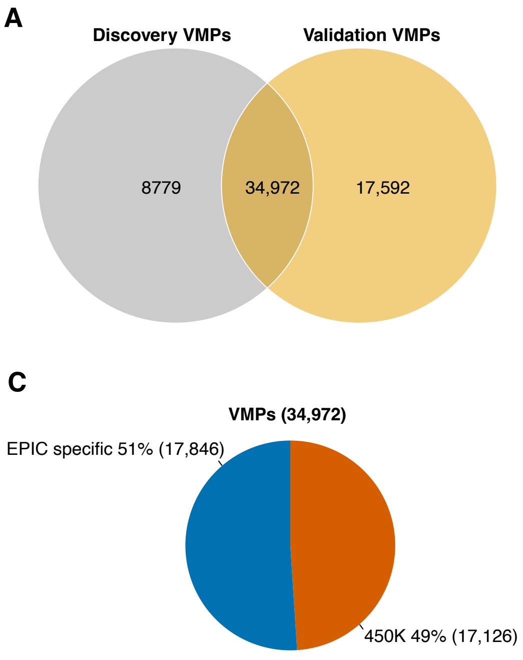

Fig. S1: *Identification of VMPs and SMPs.* Venn diagram showing the number of VMPs (A) and SMPs (B) identified in discovery and validation data sets. The percentage of VMPs (C) and SMPs (D) that are EPIC specific probes.

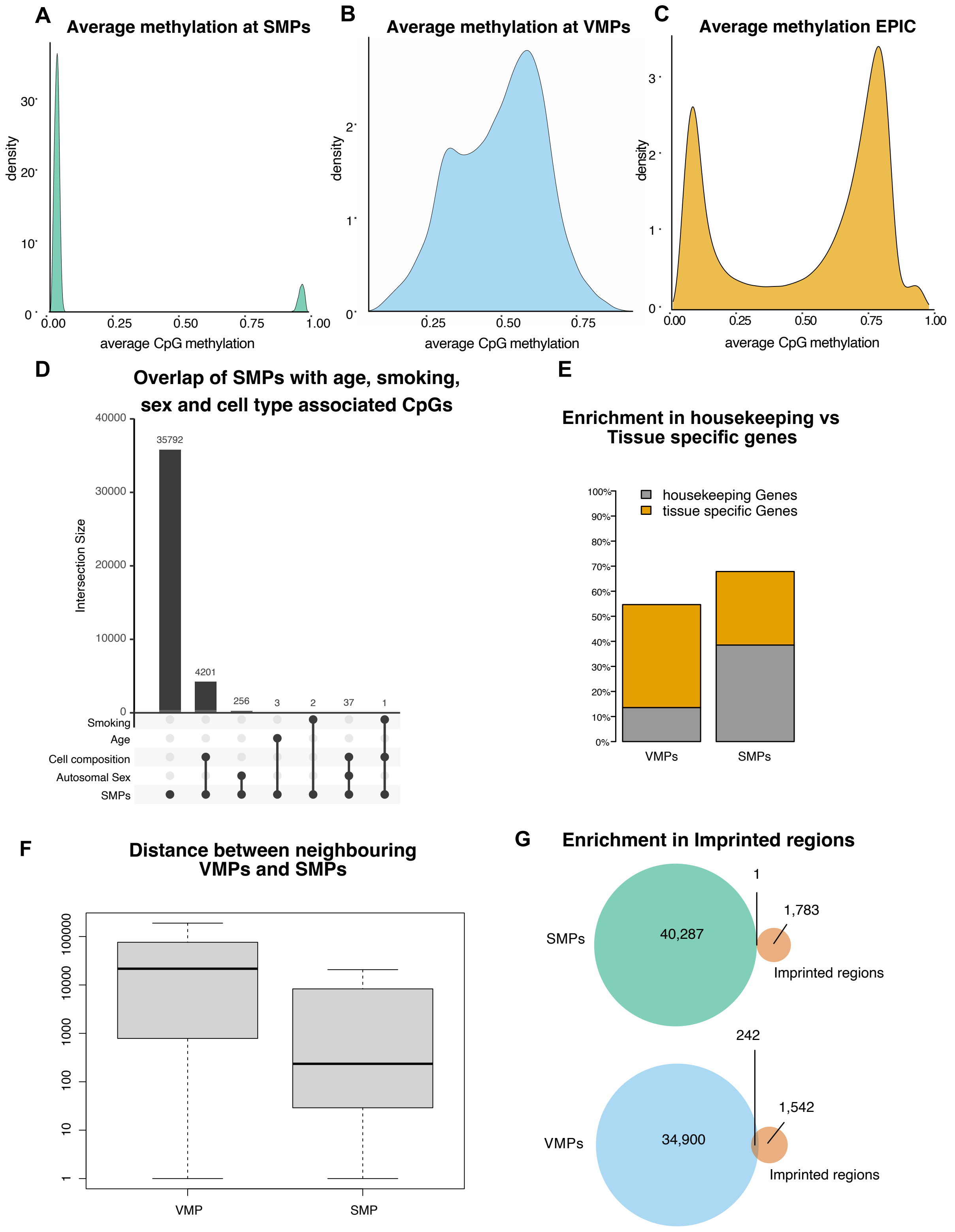

Fig. S2: *Characterisation of VMPs and SMPs.* Density plots showing distribution of average CpG methylation at (A) SMPs, (B) VMPs and (B) EPIC packground. (D) Upset plot displaying the number of SMPs that overlap with known autosomal sex, smoking, cell type and age associated CpGs. (E) Barplot representing the enrichment of VMPs and SMPs in housekeeping and tissue specific genes. (F) Boxplot of the distance between neighbouring VMPs or SMPs. (G) Venn diagrams representing enrichment of SMPs and VMPs in imprinted regions.

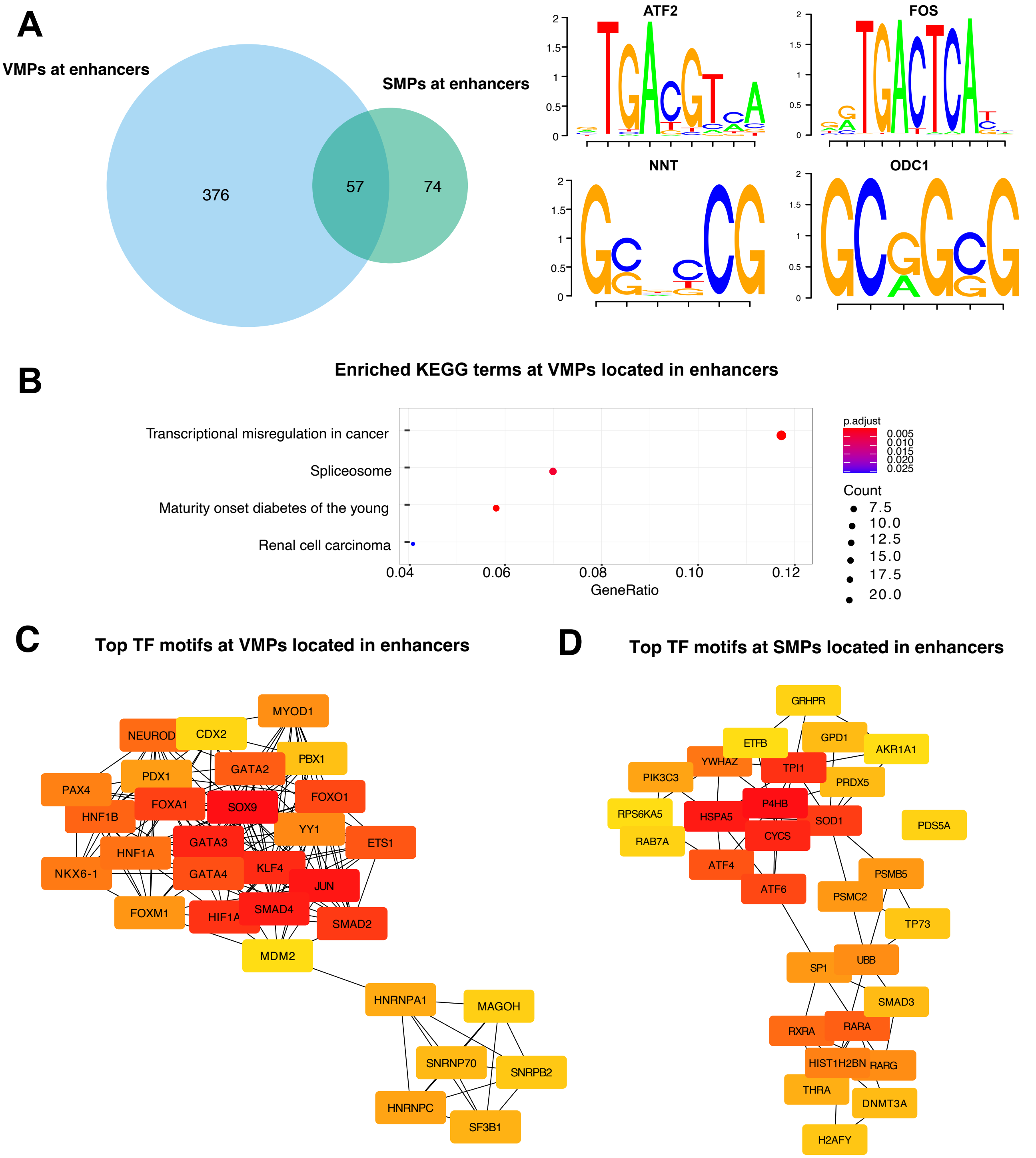

Fig. S3: *Transcription factor motif enrichment for VMPs and SMPs at enhancers.* (A) Overlap of enriched TF motifs for VMPs (blue) and SMPs (green). The top two motifs enriched in VMPs were ATF2 and FOS and in SMPs were NNT and ODC1. (B) KEGG analyses for the significantly enriched TF motifs at VMPs and SMPs at enhancers. (C-D) Sub networks of the top 30 enriched TF motifs at (C) VMPs at enhancers and (D) SMPs at enhancers. Node colour represents the degree of connectivity. The scale from red to yellow represents the top 30 enriched TF motif rank from 1-30, with red indicating highest degree and yellow indicating lowest degree.

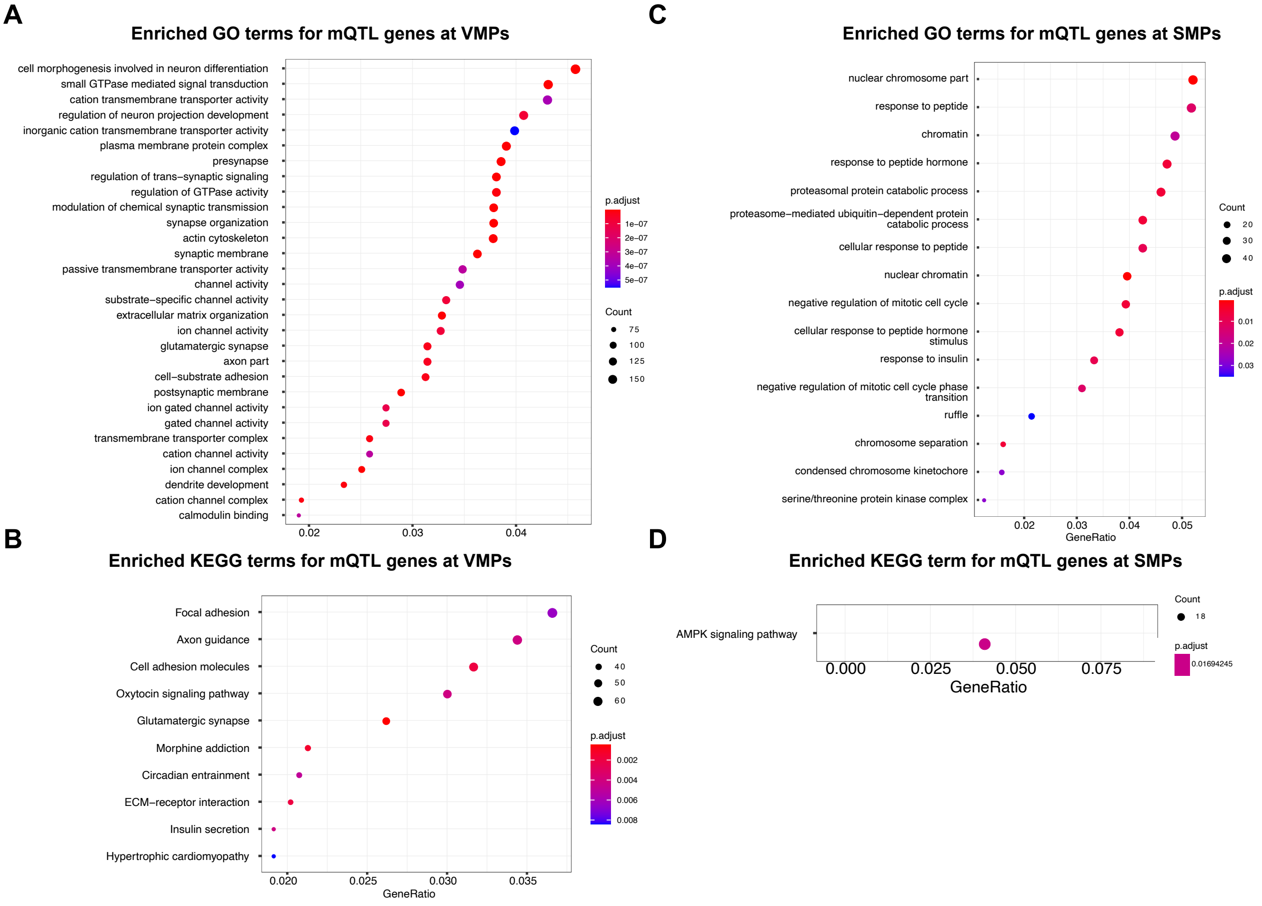

Fig. S4: *Enriched GO and KEGG terms for mQTLs.* (A) GO and (B) KEGG analyses for the mQTL genes at VMPs. (C) GO and (D) KEGG analyses for the mQTL genes at SMPs.

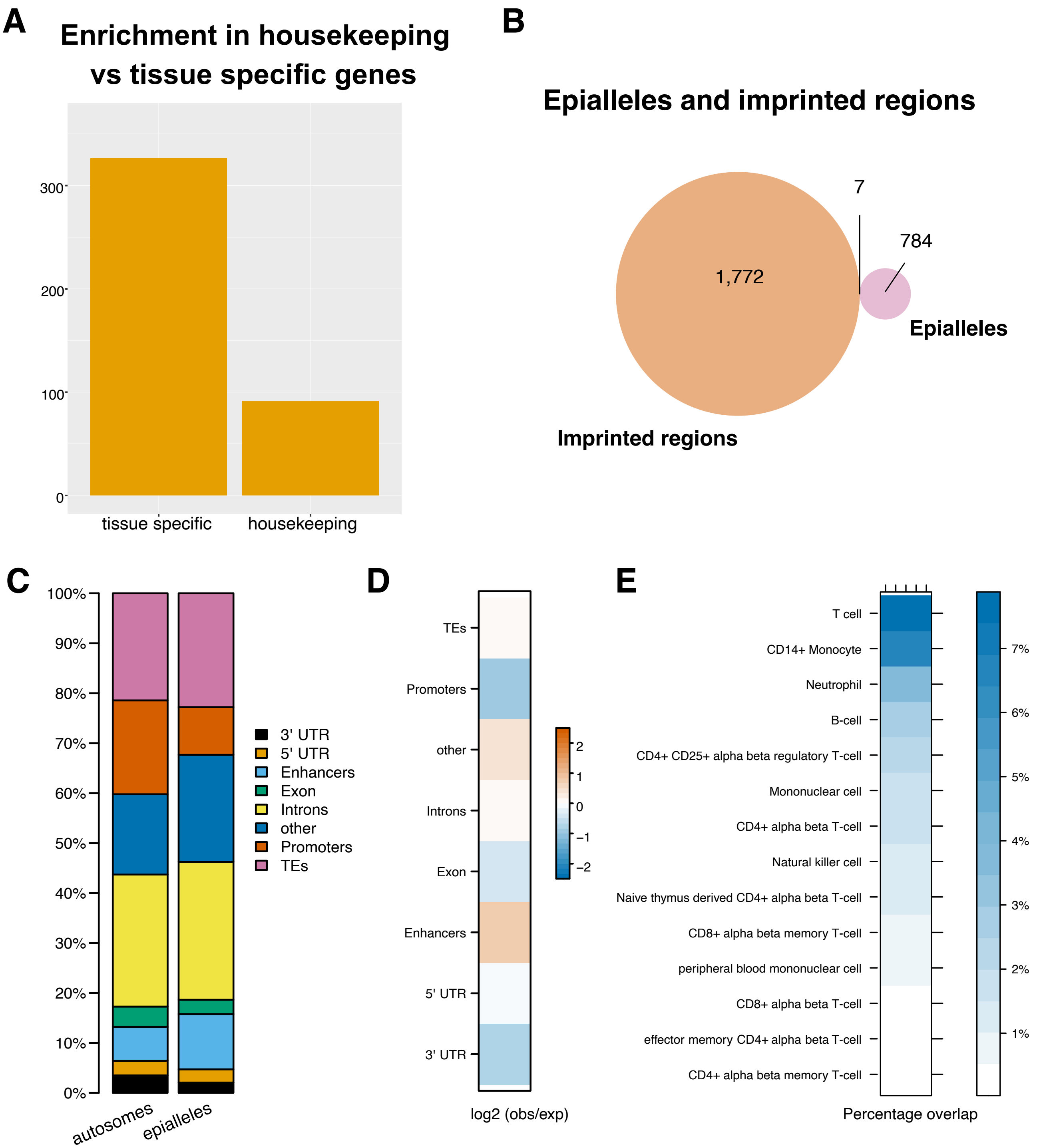

Fig. S5: *Functional annotations of putative epialleles in human whole blood.* (A) The overlap between putative epialleles and housekeeping or tissue specific genes. (B) The overlap between putative epialleles and imprinted loci. (C) The overlap of all putative epialleles (n=784) with genomic features compared to the background. (D) The $log_2(obsserved/expected)$ based on the background of the different annotations. (E) The percentage of epialleles that overlap with H3K27me3 peaks in several blood cell lines.

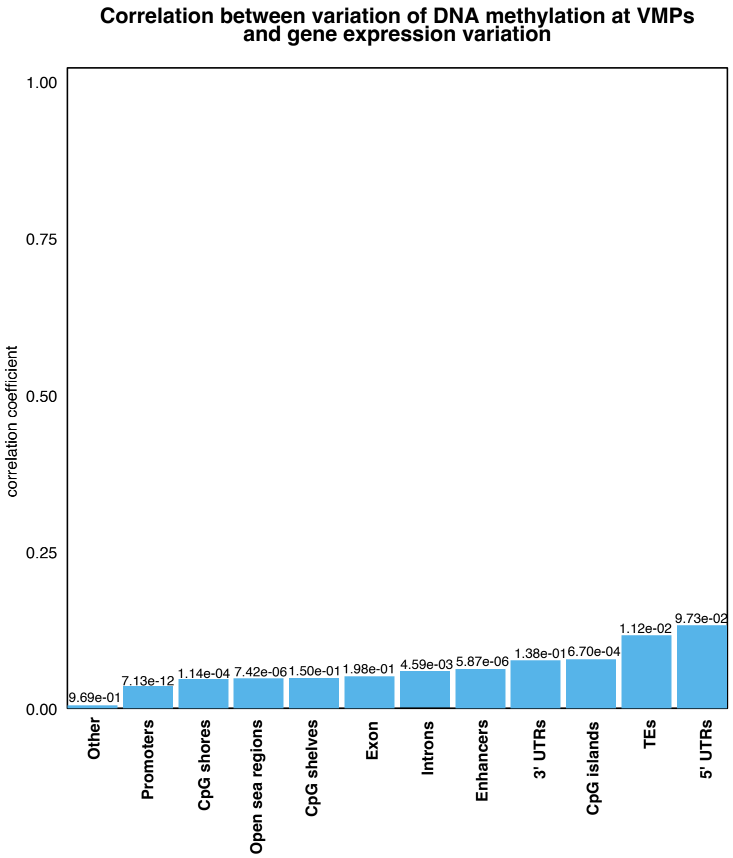

Fig. S6: *Link between methylation and expression at VMPs.* The correlation between variation in gene expression and variation in methylation of VMPs. The correlation test significance is reported above the bars.

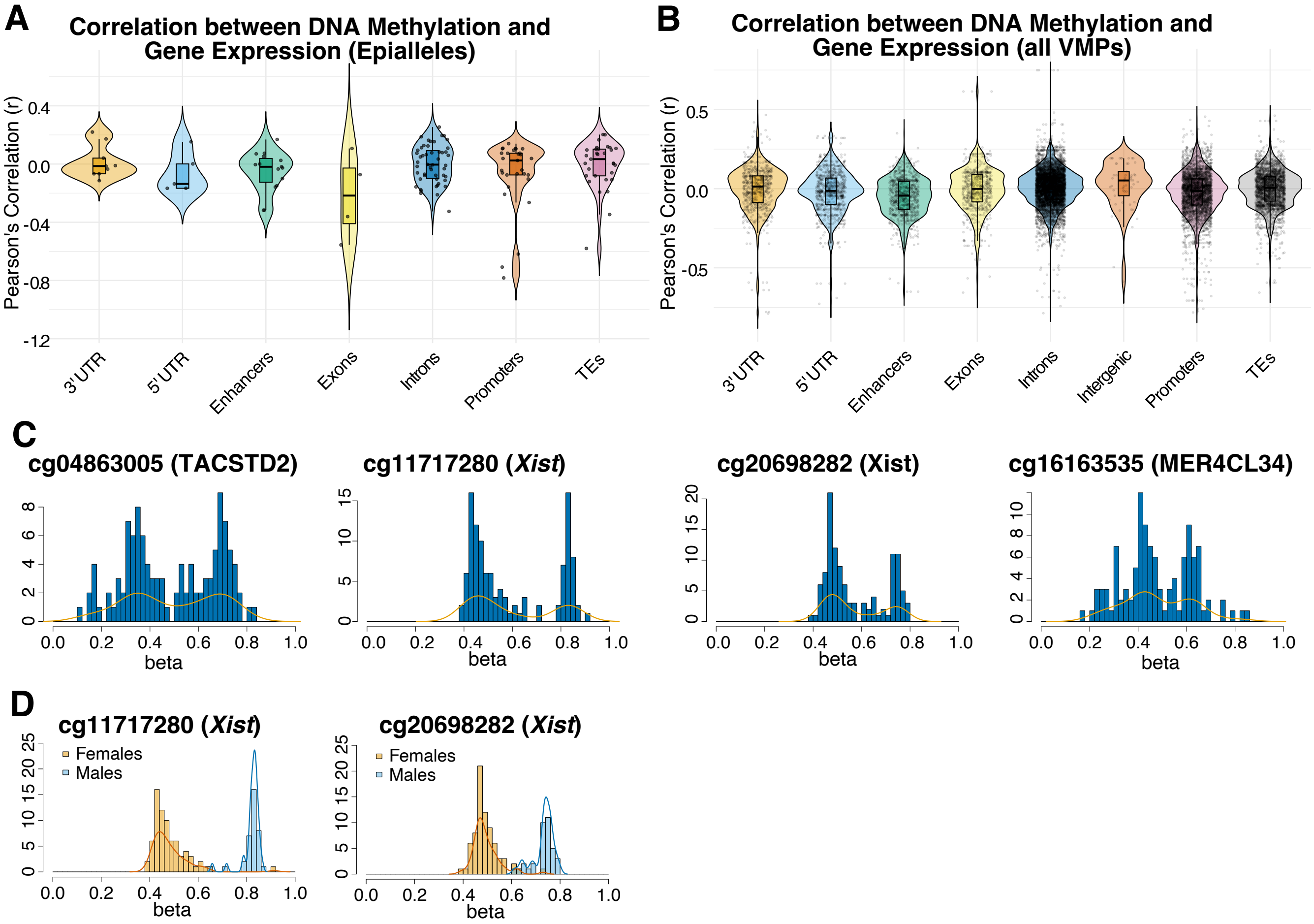

Fig. S7: *Correlation between DNA methylation and expression at epialleles and VMPs in matched samples.* (A-B) The correlation between DNA methylation levels in and gene expression levels at (A) epialleles and (B) VMPs. (C) Beta values for the four epialleles that show strong negative correlation with expression in matched samples. (D) Beta values split between males and females for the two Xist CpGs from (C).

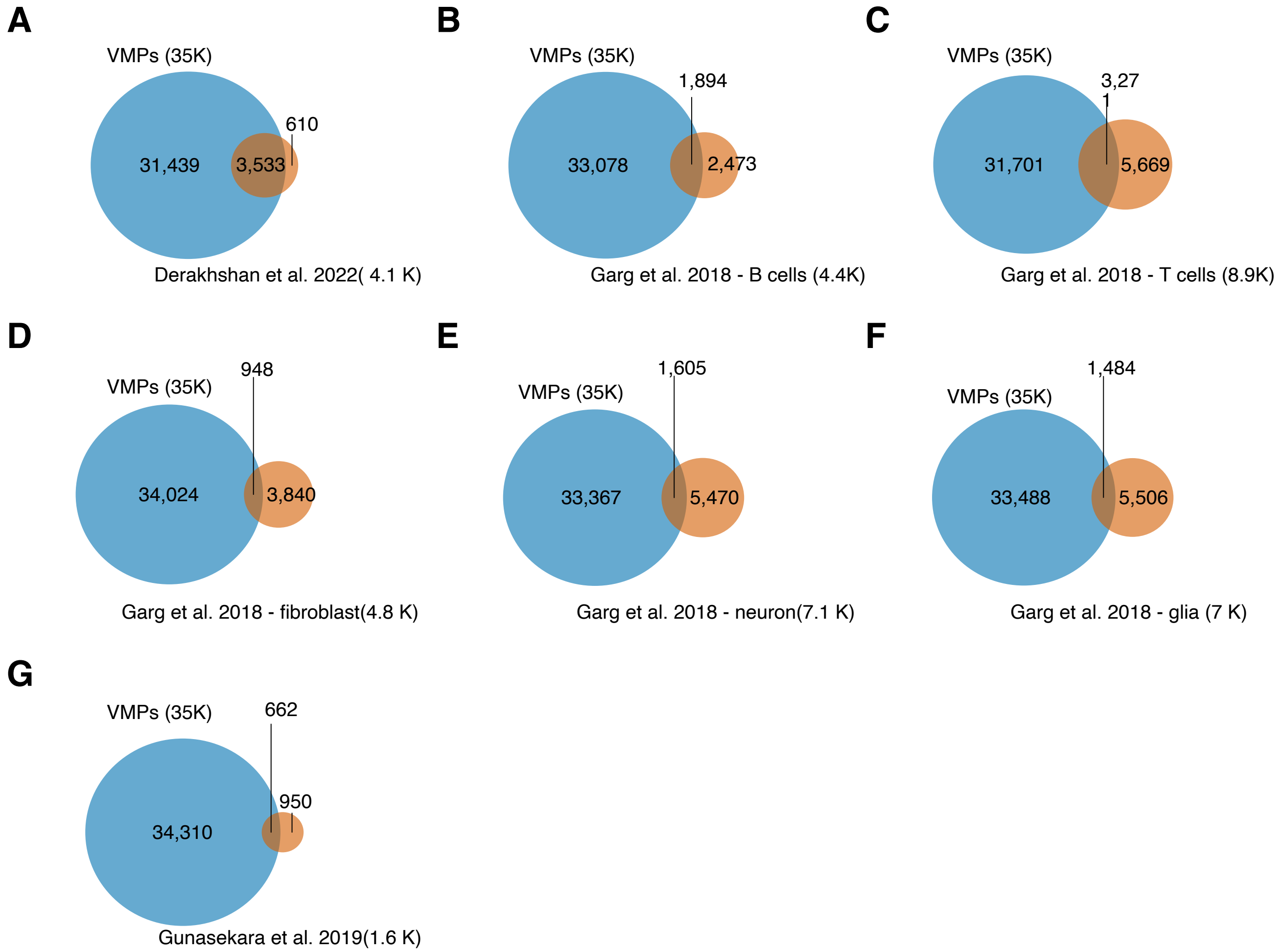

Fig. S8: *Overlap of VMPs with CpGs identified to display variable interindividual methylation in other studies.* Venn diagram showing the number of VMPs that overlap with variable probes identified in previous studies: (A) multiple tissues in [17], (B) B cells in [14], (C) T cells in [14], (D) fibroblast cells in [14], (E) neurons in [14], (F) glia in [14] and (G) multiple tissues in [31]. In the latter study, the authors identified regions displaying variable DNA methylation using bisulfite sequencing and to keep make the studies comparable, we selected only the CpGs on the EPIC array that were located in those regions.
